# Relationship between appearance-related social media consciousness and beliefs about obese persons among physical education teacher candidates

**DOI:** 10.1371/journal.pone.0350094

**Published:** 2026-07-08

**Authors:** Canan Sayın Temur

**Affiliations:** Faculty of Sports Sciences, Department of Sports Management, Ankara Yıldırım Beyazıt University, Ankara, Türkiye; National Institutes of Health, University of the Philippines Manila / De La Salle University, PHILIPPINES

## Abstract

**Objective:**

This study examined whether physical education teacher candidates’ appearance-related social media consciousness and beliefs about obese persons differed by teacher candidates’ gender and years of study and whether there was a relationship between these two factors.

**Method:**

A correlational research design was employed in the study. The study involved 153 physical education teacher candidates. Data were collected using the Demographic Information Form, the Appearance-Related Social Media Consciousness Scale and, the Beliefs about Obese Persons Scale. The data were analyzed using the Mann-Whitney U, Kruskal-Wallis, and Spearman’s rank correlation tests.

**Results:**

The results showed that there was no statistically significant difference between the level of appearance-related social media consciousness and the level of beliefs about obese persons between female and male physical education teacher candidates. In terms of years of study, the level of appearance-related social media consciousness was lower among those in their 2^nd^ and 3^rd^ years than among those in their 4^th^ year, and the level of attitudes towards obese persons was lower in the 3^rd^ year than in the 4^th^ year. The results further showed no correlation between the levels of appearance-related social media consciousness and attitudes towards obese persons among the physical education teacher candidates.

**Conclusion:**

These findings highlight the importance of physical education teacher education programs actively addressing appearance-related social media consciousness and beliefs about obese persons, with the aim being to mitigate the emergence of weight-based stigma and foster more inclusive, informed attitudes.

## Introduction

Throughout history, people have often compared themselves against ideals presented by public figures such as actors, actresses, and models in various forms of traditional media, the most common of which are newspapers, magazines, and television [1]. In contemporary society, alongside these conventional comparisons, people construct and present their images through social media platforms. This shift has led to a range of psychological issues related to self-image and appearance and has consequently become a key area of research in today’s society [2]. The increasing prevalence of social media refers to the use of online platforms that allow individuals to engage in real-time or delayed interactions with both extensive and targeted audiences. These interactions are largely based on the content generated by the users themselves, and these platforms are valued for the perceived sense of engagement with others. The importance of focusing on this particular subject is demonstrated by the current huge amount of social media use by society [3]. According to the 2024 social media usage statistics report, there are over five billion active users across various social media platforms, including Facebook, YouTube, Instagram, and TikTok. The report also predicts an increase to over six billion users by 2028, indicating a significant increase in social media adoption. Furthermore, the average daily social media usage worldwide is expected to exceed two hours, highlighting the pervasive influence of these platforms on global communication and connectivity [4].

Researchers have paid close attention to the steady increase in social media usage, highlighting the significant amount of time people spend on various social media platforms worldwide. This trend has prompted a deeper exploration of the potential advantages and drawbacks of social media, as experts aim to better understand the impact of extensive social media engagement [5]. Unlike traditional media, which predominantly displays images of others, social media platforms, where users display themselves, have a significant impact on how users perceive their appearance. This is primarily because users curate a carefully crafted representation of themselves, showcasing what they deem the most attractive, happiest, or beautiful aspects of their lives. Consequently, users often compare their appearance to selectively presented images of others on social media. Moreover, constant exposure to content and comments about appearance has amplified the significance of “body image” as a central issue within social media’s effects, encompassing both its benefits and harms [2,6].

Body dissatisfaction refers to the negative perception, thoughts, and feelings that an individual has about their own body, and this dissatisfaction stems from a perceived misalignment between an individual’s actual body image and an idealized notion of physical appearance [7,8]. This feeling arises when individuals perceive their body size and shape as inconsistent with societal standards, regardless of whether their actual appearance meets these ideals [9]. Literature emphasizes that body image is greatly influenced by social experience, and it is not fixed; it can change over time. Exposure to media messages and their perceived importance can trigger these changes. Sensitivity to such messages may also vary based on characteristics such as sex and age [7]. Social Cognitive Theory provides understanding of the impact of social media on how people see themselves [10]. This theory states that people learn how to behave and what is considered attractive by watching others. The existence of idealized bodies as models on social media allows for the learning from and comparison of users to these bodies through a process of continuous observational learning.

Numerous studies have demonstrated that excessive use of social media can have a negative impact on mental health. This is often due to pressures related to peer comparison, body dissatisfaction, feelings of sadness and stress, negative moods, low self-esteem, and a heightened risk of depression and social isolation [5,11,12]. Research has explored the connection between excessive social media use for appearance-related communication and body image, showing correlations between the frequency with which people compare their appearance on social media and their feelings of body dissatisfaction and desire for a thinner physique [2,13]. Body dissatisfaction is especially associated with social media use among young people—particularly women [6,14,15]. Moreover, this association persists across different age groups, with women showing greater susceptibility to the detrimental effects of social media on body dissatisfaction [16]. The negative effects of social media use on body dissatisfaction have also been revealed among adult women and men of various ages [17]. For instance, Marques, Paxton, McLean, Jarman, and Sibley found in a New Zealand study a link between excessive social media use and body dissatisfaction among adults [16]. Research on youth also shows that increasing social media engagement intensifies body dissatisfaction in both young women and men, often alongside idealization of thinness and muscularity [18]. A large UK mental health study in 2019 found that among teenagers aged 13–19, 40% were worried, 37% upset, and 31% embarrassed about their body image. Unrealistic “ideal” bodies portrayed in television, magazines, and social media were cited as causes for body dissatisfaction [19].

The appearance-related social media consciousness is described as a heightened awareness of one’s appearance, influenced by pervasive body image messages, particularly on photo-centric social media platforms [20]. This consciousness has a considerable adverse impact on mental health and overall well-being. The appearance-related social media consciousness reflects the extent to which individuals’ thoughts and behaviors are shaped by concerns about how attractive they appear to their social media audience, including efforts to curate an online presence aligning with societal beauty standards for validation [21]. To prevent mental health risks linked to body dissatisfaction on social media, maintaining a healthy awareness of appearance-related influences is crucial. Recent national studies report significant correlations between increased consciousness of appearance on social media and negative body image perceptions, social media addiction, and frequency and duration of social media platform use [22–24]. An international review noted that increased social media engagement correlates with greater appearance-related social media consciousness regardless of gender [25]. In the U.S., many young women exhibit a heightened appearance-related social media consciousness, which is linked to adverse effects on their body image and mental health, underscoring the need for a deeper understanding of the impact of social media on the well-being of young people [20].

In Western societies, social media messages often emphasize body dissatisfaction by promoting thinness as an ideal for women and a thin yet muscular physique for men [26]. Thinness is frequently associated with happiness, success, youth, and social acceptance, while being overweight is stigmatized with laziness, lack of willpower, lack of control, and unattractiveness [7,27,28]. These societal ideals often lead to discrimination against those not meeting them, attributing responsibility for body weight to the individual [29]. The media also plays a role in promoting negative attitudes towards overweight individuals, fostering anti-fat bias [30]. Studies report increasing prevalence of such negative beliefs globally [31,32]. Social media users who are continuously exposed to ideals of muscular or slender beauty may internalize these ideas. One important mechanism in both weight stigma models and social comparison theory is the internalization of appearance ideals, which could account for how exposure to idealized images fuels negative attitudes toward people with larger bodies as well as body dissatisfaction [18,33,34]. Stigma internalization, a theory by Durso and Latner, explains how individuals absorb and internalize negative attitudes towards obesity, often magnified through social media [35]. Idealized body images often influence public opinion, leading to individuals viewing body weight as a sign of moral character or personal worth. This internal conflict can lead to increased prejudice towards those who don’t conform to body ideals, fostering a culture of judgment and exclusion that perpetuates feelings of shame and inadequacy.

National and international research has examined beliefs about overweight individuals across populations. For example, nursing students in Turkey hold moderate beliefs, tending to view obesity as controlled by individuals [36]. In contrast, a study by Tüzün, Akgül, Işıklı, Taş, and Kambur found nurses more likely than physicians to believe obesity is not entirely under personal control [37]. Among physiotherapy students and certified practitioners, students showed stronger beliefs that obesity is not solely under an individual’s control, with male certified physiotherapists showing stronger belief in this than female physiotherapists [38]. The pervasive influence of social media shapes individuals’ emotions, thoughts, and behaviors relating to physical appearance and perceptions of those who do not fit societal body ideals [26], highlighting an important area for research given obesity’s global health threat. Rosenstock’s Health Belief Model, developed in 1974, explains how individuals’ perceptions of health, physical appearance, and personal accountability are influenced by perceived threats, advantages, and levels of control [39]. Concerns are triggered by factors like obesity, while thinness is often seen as a measure of improved health. Ideal body types, such as thinness and muscularity, are seen as benchmarks for self-discipline and vitality, while obesity is viewed as a manageable issue that indicates a lack of personal responsibility. These viewpoints are reinforced by media messaging and a societal focus on appearance, shaping a public discourse on body image and health, which can significantly influence individual beliefs and behaviors [40].

The World Obesity Federation projects that over 1 billion adults will be affected by obesity by 2030 [41]. In response, physical education (PE) is increasingly being incorporated into educational curricula as a key strategy in obesity prevention, alongside healthy nutrition [42,43]. PE teachers face the challenge of instructing young people who are heavily exposed to media ideals that highlight thinness or muscularity. Many of these young people experience body dissatisfaction and negative attitudes toward overweight individuals. It is therefore crucial that PE teachers encourage their students to adopt positive attitudes and behaviors towards diverse body types, including those who are overweight or obese [44]. Given that PE is a body-centered discipline, educators have a responsibility to prevent negative discourse and behavior among students [45]. Teachers have an important role in this regard, so it is essential that their status in relation to this issue be clarified before they are appointed to the profession and that the necessary measures be taken and regulations be put in place. In addition, understanding the dynamics of the aforementioned psychological constructs has the potential to clarify the impact of PE on the well-being and social life of young people in schools [46]. Increasing the awareness of PE teacher candidates, as well as teachers, on psychological constructs such as body perception, attitudes, and behaviors is of critical importance for the effectiveness of education in this area. By recognizing their own perceptions and prejudices, prospective teachers can develop strategies to develop positive body images in students [44,47,48]. Additionally, teacher trainees’ positive attitudes towards body diversity are important in encouraging students to accept their bodies and develop healthy lifestyle habits [49]. In this context, it is essential that body positivity, anti-prejudice pedagogical approaches, and inclusive educational strategies are integrated into PE teacher training programs [50]. Studies examining the psychological structures of prospective teachers provide guidance in understanding the attitude changes that occur during the educational process and the indirect effects of these changes on students. For example, reducing teacher candidates’ negative attitudes towards the body contributes to reducing students’ risks of social exclusion and body dissatisfaction [51,52]. However, it is also important for prospective teachers studying body-centered disciplines, such as PE and sports, to critically evaluate the effects of media on body perception [44,53]. Consequently, it is vital to assess the body-related perspectives of potential educators to enhance the inclusivity and efficacy of their future PE classes.

Considering the above literature, this study was designed to test whether PE teacher candidates differ in their appearance-related social media consciousness and beliefs about obese persons based on gender and years of study. Moreover, it aims to examine whether a significant relationship exists between these two constructs.

## Materials and methods

### Research design

Using a cross-sectional correlational comparative survey model, a quantitative research method, this study aimed to examine whether PE teacher candidates’ levels of appearance-related social media consciousness and their beliefs about obese people differ according to gender and years of study. Additionally, it sought to explore the relationship between these two constructs. The correlational comparative research model enables the analysis of group differences as well as the investigation of potential relationships between variables without manipulating them [54]. However, the study’s cross-sectional methodology restricts its capacity to draw causal inferences, as it does not account for potential confounding variables such as individuals’ body mass index (BMI), physical activity level, dieting history, or frequency of social media use. These limitations should be noted when interpreting the results.

### Participants

The study participants consisted of undergraduate students aged 18 years or older enrolled in the Department of Physical Education at the Faculty of Sports Sciences at Çukurova University, Adana, Türkiye. A total of 153 PE teacher candidates participated in the study, of whom 51% were female and 49% were male. Regarding years of study, 23.25% were 1^st^ year students, 26.8% were in their 2^nd^ year, 26.8% in their 3^rd^ year, and 22.9% in their 4^th^ year. The mean age of the participants was 21.62 (±2.68) years, with 21.23 (±1.52) years for female students and 22.03 (±3.46) years for male students. Participants were recruited between 15 May 2024 and 5 August 2024 through convenience sampling based on their the accessibility to the researcher. Prior to participation, all participants were informed about the purpose and procedures of the study, and written informed consent was obtained from all participants. This context-specific sample may reflect characteristics unique to this university population, which may introduce selection bias and limit the generalizability of the findings. As the convenience sampling method does not ensure representativeness, the results should be interpreted with caution when considering the broader population of PE teacher candidates.

### Data collection tools

The data collection instruments used in the study were a Demographic Information Form, the Appearance-Related Social Media Consciousness Scale, and the Beliefs about Obese Persons Scale.

### Demographic information form

PE teacher candidates completed a Demographic Information Form designed by researchers to collect basic background data, including age, gender, and years of study. However, the form did not include several potentially important variables, such as BMI, time spent on social media, and personal or social experiences with obesity. Including these variables as covariates in future studies could enhance the depth and accuracy of the findings by accounting for factors that may influence the outcomes.

### The appearance-related social media consciousness scale

The Appearance-Related Social Media Consciousness Scale, developed by Choukas-Bradley, Nesi, Widman, and Galla to measure the extent to which individuals are aware of and concerned about their appearance in the context of social media, including how frequently they think about, monitor, and regulate their physical appearance when using social media platforms [21], and adapted to Turkish culture by Öngören, Durdu, Dongaz, Bayar, and Bayar [55]. The scale has demonstrated strong psychometric properties, including construct validity and internal consistency, in both its original development and Turkish adaptation studies conducted with adolescent and young adult samples. Given that the present study sample consists of university-level physical education teacher candidates within a similar age range, the scale is considered appropriate for assessing appearance-related social media consciousness in this population. Some sample items from the single-dimension scale include “My attractiveness in pictures is more important than anything else I do on social media,” “If an unattractive picture of me is posted on social media, I feel bad about myself,” and “Before posting pictures on social media, I crop them or apply filters to make myself look better.” The scale consists of 13 items and is presented on a 7-point Likert scale (Never-1, Almost never-2, Rarely-3, Sometimes-4, Often-5, Almost always-6, and Always-7). The minimum and maximum scores obtained on the scale vary between 13 and 91. High scores on the scale indicate a higher level of appearance-related social media consciousness. In the original study developed by Choukas-Bradley, Nesi, Widman, and Galla, the Cronbach’s alpha reliability coefficient of 0.95 was obtained [21], while in Öngören, Durdu, Dongaz, Bayar, and Bayar’s study, during adaptation to Turkish culture, a Cronbach’s alpha reliability coefficient of 0.94 was recorded [55], and a reliability coefficient of 0.94 was calculated in this study. This finding indicates that the scale maintained a high level of internal consistency in the current sample.

### The beliefs about obese persons scale

The Beliefs about Obese Persons Scale, which was developed by Allison, Basile and Yuker to assess individuals’ underlying beliefs about the causes of obesity, specifically whether obesity is attributed to controllable personal factors such as lack of willpower and poor lifestyle choices or to uncontrollable factors such as genetics, biological predispositions, or environmental influences [29], and adapted to Turkish culture by Dedeli, Aybarc Bursalioglu, and Deveci [56]. Researchers have widely used the scale with different populations and it has demonstrated acceptable levels of construct validity and internal consistency in both its original development and Turkish adaptation studies conducted with adult and student samples. Given that the present study sample consists of university-level physical education teacher candidates, it is considered appropriate to use the scale to assess beliefs about obesity within this population. The single-dimension scale includes the following sample items: “Obesity is usually caused by overeating,” “Most obese people cause their problem by not getting enough exercise,” and “Obesity is rarely caused by a lack of willpower.” The scale was prepared as an 8-item, 6-point Likert-type scale containing positive and negative belief statements. The items of the scale were answered on a scale ranging from strongly disagree “-3” to strongly agree “+3”. Items 1, 3, 4, 5, 6, and 8 were reverse-coded. Scores were calculated by summing all 8 items and adding 24 to shift the total score range from −24–24–0–48, ensuring all scores are positive. A strong belief that obesity is not under the control of an obese person is indicated by the high mean total score obtained from the scale. During the process of developing the scale, Allison, Basile and Yuker calculated the reliability coefficient of the scale to be 0.82 [29], and Dedeli, Aybarc Bursalioglu, and Deveci calculated the Cronbach alpha reliability coefficient of the scale to be 0.84 [56] while adapting to Turkish culture, and in this study, the reliability coefficient was calculated as 0.57. This value is lower than those reported in previous studies, indicating reduced internal consistency. A lower alpha may reflect greater measurement error and attenuate correlations with other variables. Therefore, results obtained from this scale should be interpreted with caution, and this limitation is acknowledged.

### Data collection procedures

The faculty and department from which the data were obtained officially authorized the data collection, as did the research ethics committee of the Cukurova University Faculty of Medicine (approval number 144−53, approval date 10/05/2024). After obtaining approval and permission, the appropriate courses for the PE teacher candidates were determined, and with the approval of the course instructor, the data collection tools were distributed to the PE teacher candidates in a face-to-face setting, where they were informed about the study and given the information that participation was voluntary. The demographic information form and the scales were completed in about 10 minutes.

### Data Analysis

The Kolmogorov-Smirnov test was performed and showed that the distribution of the data derived from the Appearance-Related Social Media Consciousness Scale and the Beliefs about Obese Persons Scale deviated significantly from normality p < .05. Non-parametric tests were used based on this result. While the use of non-parametric tests (Mann-Whitney U, Kruskal-Wallis, and Spearman’s rho) is appropriate given the non-normal distribution, the analysis was limited to bivariate comparisons. This limits the depth of the statistical analysis.

In analyzing the data obtained in the study, the Mann-Whitney U test was used to determine whether PE teacher candidates’ appearance-related social media consciousness and beliefs about obese persons differed by gender, the Kruskal Wallis test was used to determine whether PE teacher candidates’ appearance-related social media consciousness and beliefs about obese persons differed by years of study, and Spearman’s correlation coefficient was used to determine whether there was a relationship between PE teacher candidates’ appearance-related social media consciousness and beliefs about obese persons. Dunn’s test with Bonferroni adjustment was used for post-hoc analysis following a significant Kruskal-Wallis test. The significance level was considered as p < .05 for all analyses. All statistical analyses were performed using the Statistical Package for the Social Sciences (SPSS), version 22.

## Results

The Mann-Whitney U-test was conducted to examine whether the level of appearance-related social media consciousness differed by gender between participating PE teacher candidates. The results indicated no significant gender difference (*U* = 2630.00, *p* = .281), with female and male candidates showing similar levels of appearance-related social media consciousness. The small effect size (*|r|* = 0.087) indicates a minimal practical difference (Table 1).

**Table 1:** Comparison of appearance-related social media consciousness by gender of PE Teacher Candidates.

Gender	*n*	*x̄*	*SD*	*Mdn*	*IQR*	*U*	*p*	*r*
Female	78	37.33	14.39	35.00	17.50	2630.00	.281	−.087
Male	75	34.69	13.42	34.00	17.00			
Total	153	36.04	13.94	35.00	17.25			

The Mann-Whitney U test was conducted to examine whether beliefs about obese persons differed by gender. No significant difference was found (*U* = 2919.00, *p* = .982), with female and male candidates showing similar median scores, and effect size was negligible (|r| = 0.002), indicating practically no difference between the groups (Table 2).

**Table 2:** Comparison of beliefs about obese persons by gender of PE Teacher candidates.

Gender	*n*	*x̄*	*SD*	*Mdn*	*IQR*	*U*	*p*	*r*
Female	78	19.14	6.38	18.00	8.00	2919.00	.982	−.002
Male	75	19.27	7.32	18.00	9.00			
Total	153	19.20	6.83	18.00	8.50			

The Kruskal-Wallis test was conducted to determine whether appearance-related social media consciousness differed by years of study. A statistically significant difference was found among the groups (*H* = 10.84, *p* = .013). The IQRs indicate that variability was largest in 4^th^- year candidates. Post-hoc comparisons using Dunn’s method with Bonferroni correction indicated that 4th-year candidates had significantly higher levels of consciousness compared to 2nd- and 3rd-year candidates, while differences between other pairs were not significant (Table 3).

**Table 3:** Comparison of appearance-related social media consciousness by years of study of PE Teacher candidates.

Years of Study	*n*	*x̄*	*SD*	*Mean Rank*	*Mdn*	*IQR*	*H*	*p*
1^st^ year	36	35.89	13.71	77.31	35.00	17.25	10.84	.013
2^nd^ year	41	33.29	13.10	68.34	34.00	15.50		
3^rd^ year	41	32.95	10.28	67.87	33.00	11.50		
4^th^ year	35	43.03	16.68	97.53	40.00	28.00		
Total	153	36.04	13.94					

The Kruskal-Wallis test was used to examine differences in beliefs about obese persons across years of study. A significant difference was observed among year groups (*H* = 9.83, *p* = .020). The IQRs indicate variability across groups. Post-hoc Dunn comparisons revealed that 4th-year candidates had higher belief scores than 3rd-year candidates, while other comparisons were not significant (Table 4).

**Table 4:** Comparison of beliefs about obese persons by years of study of PE Teacher candidates.

Years of Study	*n*	*x̄*	*SD*	*Mean Rank*	*Mdn*	*IQR*	*H*	*p*
1^st^ year	36	19.36	6.85	79.25	17.50	6.25	9.83	.020
2^nd^ year	41	18.37	6.90	69.27	16.00	7.00		
3^rd^ year	41	17.71	6.51	66.77	17.00	8.00		
4^th^ year	35	21.77	6.64	95.73	21.00	11.00		
Total	153	19.20	6.83					

Spearman’s correlation analysis indicated no significant association between appearance-related social media consciousness and beliefs about obese persons, with a small effect size (*r*_*s*_ = −.119, *p* = .143), suggesting minimal practical correlation (Table 5).

**Table 5:** Correlation between Appearance –Related Social Media Consciousness and Beliefs about Obese Persons Level of PE Teacher Candidates.

		Beliefs About Obese Persons
Appearance–Related Social Media Consciousness	*r*_*s*_	−.119
	*p*	.143
	*N*	153

## Discussion

The results of this study revealed no difference between appearance-related social media consciousness levels of female and male PE teacher candidates, and that these levels were similar across both groups. However, the appearance-related social media consciousness levels of PE teacher candidates in their 2^nd^ and 3^rd^ years were lower than those of 4^th^ year. In other words, ongoing concern about looking attractive to social media audiences may be elevated among 4^th^ year PE teacher candidates. Due to the limited number of studies examining appearance-related social media consciousness, particularly in the field of PE and sports and among university students, the findings were discussed in relation to studies that examined this construct as much as possible. Several national studies focusing on adolescents provide mixed findings regarding gender and years of study differences in appearance-related social media consciousness. For example, Sarman and Çiftçi reported a moderate level of awareness with no gender difference but significant variation by years of study [24]. In contrast, Çimke and Yıldırım Gürkan found that adolescent females under the age of 18 displayed higher levels of appearance-related social media consciousness than their male counterparts [22]. Internationally, Maheux, Roberts, Nesi, Widman, and Choukas-Bradley supported the observation that young female adolescents tend to have higher levels of appearance-related social media consciousness than males, further linking this to depressive symptoms in both genders [57]. Research with young adults aged approximately 18–26 also shows varied levels of appearance-related social media consciousness. Malav reported moderate awareness levels among young adults in India [58], whereas Batool and Quratulain found high levels among Pakistani young adults, with heightened consciousness being associated with social media addiction and lower self-efficacy [59]. Additionally, Akram, Ashraf, and Nouman documented a positive association between increased appearance-related social media consciousness and negative self-perception among adult women in Pakistan [60]. In studies involving university students, Ozturk and Ozturk examined fine arts students and found generally low levels of appearance-related social media consciousness, with no significant differences by gender or years of study, which partially supports the findings of the present study [23]. However, other research with university populations contradicts this, such as Özalp and Akbulut, who reported that female students exhibited higher levels of appearance-related social media consciousness than males [61]. Bratu’s review highlights that appearance-based social media behaviors—such as selfie-taking and photo editing—may reinforce cultural beauty standards and shape appearance-related beliefs regardless of individuals’ own body perceptions [62]. While this study did not observe gender-based differences in social media consciousness related to appearance, this finding should be contextualized rather than generalized. One possible explanation is that male and female PE students are exposed to similar sociocultural and academic environments, in which physical performance, health, and visual appearance gradually become more emphasized. Additionally, within PE-focused academic environments, shared norms and expectations related to fitness, body image, and physical performance could reduce the influence of gender-based differences. Moreover, the social media usage patterns and purposes might be homogenous across genders within this specific population. In addition, future PE teachers may develop a critical awareness of body ideals in media representations through the courses they take. They may also be aware of body image pressures in society and the media, which could explain their low level of social media consciousness related to appearance in both genders [63]. One possible explanation for the higher levels of appearance-related social media consciousness among 4^th^ year candidates compared to 2^nd^ and 3^rd^ year candidates could be related to changes in physical activity patterns, though this remains speculative. PE teacher candidates typically demonstrate a sufficient level of physical fitness when entering the PE teacher education program through a special talent exam. However, as they approach graduation, factors such as a reduction in practice-based courses, long hours spent studying, and preparation for teaching qualification exams may lead to weight gain and physical inactivity [64,65]. This, in turn, might heighten their sensitivity to how they are perceived on social media platforms.

The results of this study, which examined whether the level of beliefs about obese individuals differed according to the gender and years of study of the PE teacher candidates, revealed no significant difference between female and male candidates. Their beliefs about obese individuals were found to be comparable. Both female and male teacher candidates tended to believe that obesity is largely under the control of the obese individual. However, when analyzed by years of study, the level of beliefs held by 3^rd^ year PE teacher candidates was lower than that of 4^th^ year candidates, indicating that 3^rd^ year candidates are more likely to believe that obesity is under personal control compared to their 4^th^ year counterparts. Various national and international studies, involving different sample groups including those from the PE and sports fields, have yielded findings that both support and contradict the results of this study [37,66,67]. For instance, Soto, Armendariz-Anguiano, Bacardí-Gascón, and Cruz, in a study involving medical and psychology students in Mexico, reported no gender difference in beliefs about obese individuals [68]. Similarly, Lawrence, Abel, Stewart, and Dziuban found no significant gender differences among social work students in the United States [69]. Darling and Atav also reported that both male and female students believed that obesity was not entirely under the control of the individual [70], which is in line with the current study’s findings. However, contrasting results have been reported in other studies. For example, Usta, Bayram, and Altınbaş Akkaş found that female nursing students in Turkey had more negative beliefs about obese persons compared to their male counterparts [36]. Similarly, Flint, Hudson, and Lavallee, in a UK-based study, found that women were more likely than men to believe that obesity is under the control of the individual [71]. Findings regarding the years of study contrast with the findings of Vroman and Cote, who reported no significant difference in beliefs between undergraduate and graduate occupational therapy students in the United States [72]. Similarly, Usta, Bayram and Altınbaş Akkaş found no difference between nursing students’ beliefs based on years of study [36]. A limited number of studies, both nationally and internationally, have examined beliefs about obese persons and associated variables within the field of PE and sports. One of the few national studies comparing students from departments of health sciences, economics and administrative sciences, and PE found no significant difference between PE students and students from other departments; all groups held moderate beliefs that obesity is under the control of the individual [56]. Additionally, this study found no significant gender differences in beliefs about obese persons, which aligns with the findings of the current study. Internationally, Lynagh, Cliff and Morgan compared the beliefs of preservice classroom teachers who teach PE at the primary school level and specialist health and PE teacher candidates at the secondary and high school levels in Australia [73]. They found that specialist PE teachers were more likely to believe that obesity is under individual control compared to classroom teachers. Similarly, a study conducted in New Zealand compared PE and psychology students and found that PE students displayed higher levels of anti-fat prejudice than their psychology counterparts [74]. Furthermore, the same study reported that 3^rd^ year students held stronger negative stereotypes toward obese individuals than 1^st^ year students. Chambliss, Finley and Blair also identified high levels of prejudice and discriminatory attitudes toward obese individuals among exercise science students in the United States [75]. Consistent with these findings, the present study found no significant gender differences in beliefs about obese persons.

The lack of significant gender differences in beliefs about obese individuals observed in this study may be related to the shared academic and professional background of PE teacher candidates. Majors in PE and related fields often adopt the view that maintaining a healthy body—typically defined by a normal BMI—can be achieved through proper nutrition and regular physical activity. This belief, commonly reinforced in their education, may explain why both male and female participants in the current study perceived obesity as largely under individual control [76]. Supporting this, previous research has shown that both male and female fitness professionals and frequent exercisers, many of whom have no personal experience with being overweight, tend to believe that body weight is entirely controllable—an attitude shaped by their academic and cultural exposure within health and fitness contexts [77]. Similarly, PE teacher candidates may hold strong beliefs in personal responsibility due to their training, which emphasizes the promotion of physical activity and fitness among children and adolescents [78–80]. The current study did not find any gender differences in beliefs about obese individuals, which differs from some earlier research. This difference may be due to a mix of contextual, cultural, and disciplinary factors. Beliefs about obesity are shaped not just by personal attitudes but also by the academic environment and cultural norms about body image, health, and responsibility. In this study, all participants were PE teacher candidates with similar educational backgrounds that focused on physical fitness, health promotion, and personal responsibility. This shared training may have led to similar beliefs across genders. In contrast, earlier studies that found gender differences often included participants from a wider range of academic fields or cultural backgrounds, which could explain the different results. These findings suggest that beliefs about obesity and gender are not the same everywhere but depend on cultural expectations, social attitudes about weight, and academic training.

The study found that 3^rd^ year PE teacher candidates reported more negative beliefs about obese individuals than those in their 4^th^ year. In the early years of their training, the perception that obesity was controllable did not arise from knowledge gaps but from oversimplified understanding of obesity. However, by the 3^rd^ year, teacher education courses, such as those on fitness and training theory, along with the idea that fitness can result from individual effort, began to challenge this perception. Furthermore, practicum experiences with obese children likely fostered empathy and understanding, shifting beliefs in a more positive direction. These findings suggest a potential trend that PE teacher candidates may develop more informed and empathetic attitudes as they progress through their education, though causual inference is not possible. Another possible reason for this is that 4^th^ year teacher candidates have undertaken more coursework and practical experience covering the various factors behind obesity, including biological, psychological, and social influences, as well as concepts such as body diversity and holistic health. This broader academic and clinical background could be associated with less simplistic views of obesity, although the cross-sectional design prevents causual conclusions regarding whether obesity is solely seen as a matter of personal control [74,81].

It was determined by this study that no significant correlation exists between the level of appearance-related social media consciousness and beliefs about obese persons. The appearance-related social media consciousness construct examined in this study measures individuals’ awareness of their own appearance on social media, such as comparing themselves to the liked appearances shared by others or experiencing anxiety about their own photos, while the beliefs about obese persons construct measures beliefs about obese individuals—specifically, the individual’s attitude toward whether obesity is under their own control. The low social media awareness and beliefs about obese individuals found in this research, but the lack of a relationship between them, may be due to the different constructs and dimensions the scales measure. In other words, while one measures internal awareness of appearance on media and the other measures prejudice regarding obesity, these measures involve different psychosocial mechanisms. Social media consciousness is individual awareness of body image, while obesity prejudice is a prejudiced construct based on ideological, social, and professional norms. This may be one of the main reasons why no relationship was found between the two constructs in this study. In addition, while PE teacher candidates’ social media awareness is low, their prejudices against obese individuals may not be low either, because these two constructs, media/body awareness and obesity beliefs, consist of different social and psychological processes. While social media awareness stems from internalized body image and media use, the obese stereotype is shaped more by societal beliefs, ideology, and socialization. The “athletic/fit body” ideal internalized in the media may fuel obesity bias, but it is not directly correlated with awareness of social media use. Furthermore, as preservice PE teachers excessively focus on body image and physical performance during their education; they may develop an unconscious bias against obesity—a bias not directly related to social media awareness [73,74,82–84].

## Conclusion and Recommendations

The results of the study showed that the level of appearance-related social media consciousness and beliefs about obese persons did not vary by gender among PE teacher candidates but did vary by years of study. Teacher candidates of both genders exhibit comparable levels of ongoing awareness regarding their potential social media audience and biased beliefs about obese individuals. However, in terms of years of study, 4^th^ year teacher candidates reported a higher level of ongoing awareness about their appearance on social media than 2^nd^ and 3^rd^ year candidates (who reported lower scores). They also reported higher belief scores regarding obese individuals than 3^rd^ year candidates. In addition, no significant relationship was found between the level of appearance-related social media consciousness and beliefs about obese persons. In light of these findings, academic literature on body image and social media awareness has indicated that gender differences are not absolute. However, factors such as level of education or year of study impact individuals’ appearance-focused social media consciousness [21]. The relatively lower levels of appearance-related social media consciousness and biased beliefs observed in both male and female PE teacher candidates may be considered a positive indication, but this should be interpreted cautiously given the study design. However, the bias scores gathered from the PE teacher candidates, still reflect the concern that how they appear on social media and obesity is the responsibility of individuals who are obese. This mildly negative belief emphasizes the importance of physical education teacher candidates questioning and changing their perceptions and beliefs regarding their own social media appearance and that of obese individuals. This suggests that individuals’ appearance-focused consciousness and socio-cultural biases may evolve differently as they progress through education; in other words, education plays a complex and multidimensional role in shaping attitudes towards body image and social media awareness. In this context, the findings support the idea that the appearance- and body-related consciousness and attitudes of young adults should be evaluated together but independently throughout their education.

The findings of this study must be interpreted with caution, owing to several potential limitations. This study was conducted using a cross-sectional research design that collected data at a single point in time. While this approach allows for quick and practical analysis of possible relationships between variables, it is not sufficient to determine causality. Therefore, the relationships observed in this study should be interpreted only as correlations, not causality. Since cross-sectional studies do not provide the opportunity to observe individual and social changes that may occur over time, it should be taken into account that the findings obtained from this study only reflect the situation at the time of the research and do not reflect the dynamics that change over time.

In this cross-sectional study, confounding variables may have misleadingly affected the relationship between the independent and dependent variables. For example, factors not measured in this study, such as candidate physical education teachers’ BMI, whether they have an obese friend or family member, whether they do physical activity, how much time they spend on social media, which social media platforms they use, etc., may have played a role in shaping the findings as intervening variables and negatively affected the internal validity of the study.

In this study, the convenience sampling method was used; that is, participants were selected from individuals who were easily accessible. This situation should be taken into account in the interpretation of the findings, as it reduces the capacity of the study’s sample to represent the target universe and external validity. Because the sample was not randomly selected, certain subgroups may have been over- or under-represented, and findings should only be generalized to the individuals included in the sample. Generalizing it to the candidate physical education teacher population across Türkiye may be problematic.

Future research could observe changes in the appearance-related social media consciousness and beliefs about obese persons in PE teacher candidates longitudinally from 1^st^ to 4^th^ year. Furthermore, a qualitative research design could be used to examine the reasons for this change in depth. Further research could also replicate this study by examining moderator variables such as BMI, having overweight friends, and body satisfaction, as these are likely to influence the appearance-related social media consciousness and beliefs of PE teacher candidates regarding obese people. These two variables could also be examined among majors in other departments, such as coaching education, sports management, and recreation, to make comparisons.

Based on these research findings, physical education teacher training programs could incorporate media literacy and body awareness content to raise prospective teachers’ awareness of their appearance on social media and reinforce respect for body diversity. Similar content could also be incorporated into seminars for practicing physical education teachers. Furthermore, this content could be converted into digital formats and shared as digital training resources.

## Supporting information

S1 File Underlying study dataset. (SAV)
