# Supplementary material for: Relationship between appearance-related social media consciousness and beliefs about obese persons among physical education teacher candidates
Source: PLoS One. 2026 Jul 8;21(7):e0350094. doi: 10.1371/journal.pone.0350094 (PMC13345289; doi:10.1371/journal.pone.0350094)
Supplement: Attachment — Submitted filename: Response to Reviewers Comments.pdf [file pone.0350094.s005.pdf]

Dear Editor,

I sincerely thank you for your constructive feedback, support, and invaluable comments, which were of great help in revising the manuscript.

My responses to your comments are given below.

I hope with your suggestions, the manuscript will better suit the PLOS ONE Journal.

Manuscript ID: PONE-D-25-12083

Title: Relationship between appearance-related social media consciousness and beliefs about obese persons among physical education teacher candidates

Sincerely,

Canan Sayın Temur

**Reviewer's comments**	**Revisions**
1. Is the manuscript technically sound, and do the data support the conclusions?<br><br>The manuscript must describe a technically sound piece of scientific research with data that supports the conclusions. Experiments must have been conducted rigorously, with appropriate controls, replication, and sample sizes. The conclusions must be drawn appropriately based on the data presented.<br><br>Reviewer #1: Yes<br><br>Reviewer #2: Partly	I would like to express my sincere gratitude to the reviewers for their careful review and feedback.<br><br>This is a technically sound scientific study, and the data provided strongly support the reliability of the results. The study was meticulously planned with reproducibility in mind, and the sample size was determined in accordance with scientific standards. Transparency and objectivity were maintained when interpreting the results, using both quantitative data and visual tables. While Reviewer #1's positive assessment of 'Yes' confirms this, Reviewer #2's assessment of 'Partially' may indicate some shortcomings or areas requiring improvement.<br><br>A careful review of the entire research manuscript was therefore undertaken, concluding that the study is methodologically sound and that the data are robust enough to support the conclusions.
2. Has the statistical analysis been performed appropriately and rigorously?<br><br>Reviewer #1: Yes<br><br>Reviewer #2: No	Thank you for your valuable feedback regarding the statistical analysis. In response to your comment, I have revised the statistical section to enhance the rigor of our analysis. Specifically, I have calculated and reported the effect sizes for the Mann–Whitney U tests, as recommended for non-parametric comparisons. Additionally, for the Kruskal–Wallis tests that yielded statistically significant results, I have conducted post-hoc pairwise comparisons using Dunn's test with Bonferroni correction to control for multiple comparisons. These changes have been incorporated into method/data analysis and results section of the revised manuscript, and I believe they strengthen the statistical robustness of the findings.

3. Have the authors made all data underlying the findings in their manuscript fully available?<br><br>The PLOS Data policy requires authors to make all data underlying the findings described in their manuscript fully available without restriction, with rare exception (please refer to the Data Availability Statement in the manuscript PDF file). The data should be provided as part of the manuscript or its supporting information, or deposited to a public repository. For example, in addition to summary statistics, the data points behind means, medians and variance measures should be available. If there are restrictions on publicly sharing data—e.g. participant privacy or use of data from a third party—those must be specified.<br><br>Reviewer #1: No<br><br>Reviewer #2: No	Thank you for your suggestion.<br><br>I confirm that all data underlying the findings of our manuscript will be made fully available in accordance with the PLOS ONE Data Availability Policy.<br><br>The complete dataset will be provided as part of the Supporting Information files.
4. Is the manuscript presented in an intelligible fashion and written in standard English?<br><br>PLOS ONE does not copyedit accepted manuscripts, so the language in submitted articles must be clear, correct, and unambiguous. Any typographical or grammatical errors should be corrected at revision, so please note any specific errors here.<br><br>Reviewer #1: Yes<br><br>Reviewer #2: Yes	I thank both reviewers for their careful assessment and positive feedback regarding the manuscript's clarity and use of standard English (Reviewer #1: Yes; Reviewer #2: Yes).<br><br>I have thoroughly revised the manuscript from beginning to end, focusing on language, grammar, and typographical accuracy to ensure that the text is as clear, correct, and unambiguous as possible.<br><br>All identified errors have been addressed in the revised version.

Reviewer #1: This is a thoughtfully executed study on a topic of growing global relevance. It successfully addresses a research gap, contributes valuable cross-sectional data, and lays groundwork for further exploration into stigma and digital self-awareness in teacher education. While the analyses are basic and the conclusions somewhat cautious, the manuscript offers timely insights that merit dissemination. Observations and opportunities to improve the paper are as follows:<br><br>Introduction<br><br>The manuscript's objectives are clearly stated and relevant, particularly considering the WHO's projections on obesity prevalence and the pedagogical imperative for inclusive education. However, the introduction could benefit from a sharper statement of the core research question, ideally summarized at the end of the first section to enhance coherence.<br><br>Recommendation:<br><br>Clarify and explicitly state the research question/s at the end of the Introduction.<br><br>Condense the literature review to reduce redundancy and improve flow.	I would like to sincerely thank both reviewers. Their feedback was insightful and constructive. I have thoroughly revised the introduction section in response to the following points that were raised. I have rephrased the purpose statements at the end of the first section of the Introduction to make them clearer and more concise. This enhanced coherence and provide readers with a more focused understanding of the study's aims.<br><br>I have also made improvements to the literature review. I have removed references that are no longer needed and restructured the narrative to make it more reader-friendly. The context was made more succinct and relevant to the research objectives as a result.

Methods<br><br>Observation:<br><br>Convenience sampling may introduce bias and limits generalizability. Cross-sectional design precludes causal inference. The study does not control for potential confounders such as BMI, personal history of dieting, or social media use frequency. Post-hoc testing lacks correction for multiple comparisons, increasing the risk of Type I error.<br><br>Recommendation:<br><br>The methods section should discuss these limitations more transparently, especially the implications of sampling bias and uncontrolled confounding variables.<br><br>Report effect sizes for all inferential tests and consider corrections for multiple comparisons.	I sincerely thank the reviewers for their valuable and constructive suggestions regarding the Methods section.<br><br>I have carefully considered your feedback and made the necessary revisions to address the concerns.<br><br>I have expanded the Method/Participants and Limitations sections to more explicitly acknowledge the use of convenience sampling and the inherent limitations it poses in terms of potential selection bias and limited generalizability.<br><br>I have also clarified that the cross-sectional design precludes any causal inferences, and I have discussed how this affects the interpretation of the findings in both the Method/Research Design and Limitations sections.<br><br>Furthermore, I have acknowledged in the manuscript that important potential confounders—such as BMI, personal history of dieting, and frequency of social media use—were not controlled for. The implications of these uncontrolled variables have been discussed as a limitation.<br><br>Effect sizes have now been reported for all inferential statistical tests to provide a more comprehensive understanding of the magnitude of observed effects.<br><br>Additionally, I have revised the statistical analysis to include corrections for multiple comparisons in all post-hoc tests. Specifically, the Bonferroni correction was applied to reduce the risk of Type I error.<br><br>These revisions are reflected in the Methods, Results, and Limitations sections of the revised manuscript.

Results/ Discussion<br><br>Observation:<br><br>The interpretations are consistent with the data and responsibly framed within the study's limitations. The authors rightly caution against overgeneralization and call for targeted training in PE curricula. Notably, they interpret the absence of gender differences and the significance of grade-level trends with nuance, acknowledging sociocultural factors and the potential role of educational exposure. However, the conclusion about "lower-than-average" beliefs and awareness is not empirically substantiated and seems speculative.<br><br>Recommendation:<br><br>Avoid drawing normative conclusions (e.g., that beliefs are "lower than average") unless benchmark data are cited. Emphasize the potential for curriculum interventions rather than assuming deficit thinking among participants.<br><br>Suggest future research directions involving longitudinal or qualitative methods to explore causality and context.	I sincerely thank the reviewers for their valuable and constructive suggestions.<br><br>I have replaced normative expressions (such as stating beliefs are "lower than average") with neutral, descriptive language. Where appropriate, I introduced benchmark data or referenced comparable studies to contextualize findings.<br><br>In the Discussion section, I have also added a dedicated paragraph outlining several future research directions, such as the use of longitudinal designs to better assess causal relationships, and the inclusion of qualitative methods to gain a deeper understanding of participants' experiences and perspectives. In addition to suggestions for future studies, I have included practical implications of the findings, offering recommendations that may inform interventions and practices in relevant applied settings.
Reviewer #2: 1. Significance and Relevance<br><br>a. The topic is highly relevant, especially in the context of increasing body dissatisfaction and obesity stigma amplified by social media exposure. The focus on future PE teachers is well-justified as they play a key role in shaping student attitudes toward physical health and body image. b. However, the manuscript misses an opportunity to deepen its theoretical and practical contributions. While the descriptive findings are useful, they are not sufficiently connected to broader frameworks such as social cognitive theory, stigma internalization, or health belief models.	I sincerely thank Reviewer #2 for highlighting the importance of deepening the manuscript's theoretical and practical contributions. We have carefully addressed the feedback.<br><br>I have enriched the Introduction by explicitly framing study findings within established theoretical models of Social Cognitive Theory (Bandura, 1986), Health Belief Model (Rosenstock, 1974), Stigma Internalization (Durso & Latner, 2008). These additions aim to enhance the manuscript's depth, ensuring it makes clearer contributions to both theory and practice.

2. Research Questions and Rationale<br><br>a. The study's objectives are clearly stated, but the rationale for exploring the relationship between appearance-related social media consciousness (ARSMC) and beliefs about obese persons (BOP) could be strengthened.<br><br>b. There is limited justification for why these two constructs should be correlated. While both relate to body image, a theoretical link (e.g., internalization of appearance ideals leading to weight stigma) would clarify expectations and better explain null results.<br><br>The manuscript might benefit from explicitly stating hypotheses or expected directions of relationships.	Thank you for the insightful comments. In response, the manuscript has been revised to strengthen the rationale for examining the relationship between appearance-related social media consciousness (ARSMC) and beliefs about obese persons (BOP). Theoretical links have been clarified, and objectives with expected directions of relationships have been explicitly stated to provide clearer guidance for the study.
3. Sample and Generalizability<br><br>a. The study sample is limited to one university and selected via convenience sampling, which restricts external validity. This should be acknowledged more critically in the discussion section.<br><br>b. A sample of 153 participants is sufficient for nonparametric analysis but may be underpowered to detect small to moderate correlations. This limitation is not mentioned.<br><br>c. The demographic profile lacks important variables such as participants' BMI, frequency/type of social media use, and personal experience with weight stigma, which could all be relevant moderators or confounders.	Thank you for these valuable comments.<br><br>I acknowledge the limitations related to the use of a convenience sample from a single university. This limitation and its implications for the external validity and generalizability of the findings have been more critically discussed in the revised limitations section.<br><br>I also recognize the absence of potentially relevant demographic variables such as participants' BMI, the frequency and type of social media use, physical activity level, and personal experiences with weight stigma. These factors may serve as important moderators or confounders. I have addressed this omission in the limitations section and discussed how inclusion of these variables could inform future research.

4. Measures and Instrumentation<br><br>a. The ARSMC and BOP scales used are both validated and their Turkish adaptations are appropriate. The reported Cronbach's alpha coefficients (0.94 and 0.85, respectively) indicate strong internal consistency.<br><br>b. It would be helpful to include more information about the factor structure or example items in the Results or Appendix for readers unfamiliar with these scales.<br><br>c. The BOP scale's scoring method (adding 24 to the sum of reverse-coded items) could be better explained, as this may not be intuitive to all readers.	Thank you for your helpful comments.<br><br>I appreciate your positive remarks regarding the validity and internal consistency of the ARSMC and BOP scales, as well as the appropriateness of their Turkish versions.<br><br>To improve clarity for readers unfamiliar with these scales, I have expanded my descriptions of both instruments in the Methods section. I have provided additional information on the content and focus of each scale. Furthermore, example items from both the ARSMC and BOP scales have been added to give readers a clearer understanding of what the instruments measure.
5. Statistical Analysis<br><br>a. The use of nonparametric tests (Mann-Whitney U, Kruskal-Wallis, and Spearman's rho) is justified due to the non-normal distribution of scores. However, the manuscript relies exclusively on bivariate analyses.<br><br>b. The absence of multivariate analyses is a missed opportunity. Simple adjustments for confounders such as age, gender, year level, or self-reported social media use could have added depth to the findings.<br><br>c. The decision not to use regression or structural equation modeling to explore mediation or moderation effects limits the interpretability of the null findings.	Thank you for your insightful comments regarding the statistical analyses.<br><br>In response, I have calculated and reported effect sizes for the Mann–Whitney U tests to provide a clearer indication of the magnitude of observed effects.<br><br>For the Kruskal–Wallis tests that yielded statistically significant results, I performed post-hoc pairwise comparisons using Dunn's test with Bonferroni correction to account for multiple comparisons.<br><br>Regarding the concern about confounding variables, I acknowledge the limitation posed by not including multivariate analyses in the current study. I have added a discussion of potential confounders in the Method and Limitations section of the manuscript, highlighting how these uncontrolled variables may impact the findings and interpretation.<br><br>While regression or structural equation modeling approaches could offer deeper insights into mediation or moderation effects, these were beyond the scope of the current exploratory analysis.

6. Results Interpretation<br><br>a. The findings are presented clearly, but the emphasis is on statistical significance rather than effect size or practical relevance. Reporting median differences, interquartile ranges, or even using boxplots could enhance the results section.<br><br>b. The null correlation between ARSMC and BOP is potentially meaningful and deserves deeper interpretation. Could it reflect a disconnect between internalized appearance concerns and external attitudes toward others?<br><br>c. The pattern of higher ARSMC and more positive beliefs among 4th-year students raises interesting possibilities. Have they received specific training that influenced their perceptions? Is there an educational progression effect?	Thank you for these thoughtful and constructive comments.<br><br>In addition to reporting statistical significance, I have now included relevant effect size measures to provide a better understanding of the practical relevance of the findings. Where appropriate, I have also incorporated median differences and interquartile ranges to enhance the clarity and informativeness of the results.<br><br>The lack of a significant correlation between ARSMC and BOP was further examined in the revised Discussion section. I now offered a more in-depth interpretation of this null finding, supported by relevant literature. Specifically, I discussed the possibility that internalized appearance concerns and beliefs about others' obesity may operate independently, as suggested in prior research.<br><br>The observed pattern of higher ARSMC and more positive BOP scores among fourth-year students has been addressed in the discussion, with consideration of possible educational or developmental influences. These interpretations are now communicated more clearly to the reader.

7. Discussion and Integration with Literature<br><br>a. The discussion offers a useful summary of national and international studies, but it lacks a critical synthesis. Many citations are used descriptively without analyzing why findings converge or diverge.<br><br>b. The interpretation of null gender differences could benefit from engagement with recent debates on gendered body image expectations, especially in athletic or PE contexts.<br><br>c. The lack of a correlation between ARSMC and BOP should not be interpreted simply as "no relationship"; alternative explanations such as measurement limitations, range restriction, or unmeasured confounding should be acknowledged.	Thank you for your valuable suggestions regarding the Discussion section.<br><br>In response, I have thoroughly revised the Discussion by incorporating additional recent literature to provide a more critical synthesis of findings rather than a descriptive summary.<br><br>New subsections have been added to engage with contemporary debates on gendered body image expectations, particularly in athletic and physical education contexts, to better interpret the null findings related to gender differences.<br><br>Furthermore, I have expanded the discussion around the lack of correlation between appearance-related social media consciousness and beliefs about obese persons.<br><br>These enhancements have strengthened the theoretical integration and interpretation of the results.
8. Limitations and Future Directions<br><br>a. Some limitations are mentioned, such as convenience sampling and small sample size, but these are treated briefly. A more reflective account of the study's methodological constraints would strengthen the manuscript.<br><br>b. The recommendation to revise PE curricula is reasonable but would benefit from greater specificity. For example, what pedagogical approaches or training modules are proposed? Have such interventions been tested elsewhere?<br><br>c. Future research should consider a longitudinal design to examine how PE candidates' beliefs evolve over time and how professional training mediates these changes.	The manuscript acknowledges certain methodological limitations, including the use of convenience sampling and a small sample size. Although initially addressed briefly, the limitations has been expanded to provide a more reflective and critical account of these constraints, emphasizing their potential impact on the study's generalizability and validity.<br><br>The recommendation to revise PE curricula is reasonable and has been elaborated with suggestions for incorporating various specific courses and training modules within PE teacher training programs and PE in-service training modules.

9. Writing Style and Structure<br><br>a. The manuscript is generally well-organized and readable. However, the introduction and discussion are quite long and at times repetitive. Several paragraphs could be condensed to improve focus and clarity.<br><br>b. Some terms (e.g., "beliefs about obese persons") are used frequently without variation; editorial revision could enhance linguistic variety and readability.<br><br>c. The manuscript would benefit from professional language editing to tighten the narrative, especially in the background and discussion sections.	Thank you for the valuable feedback. In response to the suggestions, the manuscript has been carefully revised to improve organization and readability. The introduction and discussion sections, which were previously lengthy and occasionally repetitive, have been condensed by restructuring several paragraphs to enhance focus and clarity.<br><br>The manuscript has been carefully reviewed from a language perspective, with necessary revisions made to improve linguistic variety and overall readability. Repetitive terms have been varied, and the narrative has been tightened, particularly in the background and discussion sections.
10. Ethics and Data Availability<br><br>a. Ethical approval is clearly stated, and participant consent was obtained, which aligns with ethical standards.<br>b. The data availability statement ("available upon reasonable request") falls short of PLOS ONE's expectations. Authors should consider uploading anonymized datasets to a public repository or providing more detailed access instructions.	Thank you for your suggestion. As mentioned above I confirm that all data underlying the findings of our manuscript will be made fully available in accordance with the PLOS ONE Data Availability Policy.<br><br>The complete dataset will be provided as part of the Supporting Information files.
Overall Recommendation: Major Revisions<br><br>This manuscript addresses an important and socially relevant issue, using validated instruments and appropriate analysis. However, the study design is limited in explanatory depth, and the theoretical integration is underdeveloped. With substantial revisions—particularly in expanding interpretation, integrating theory, and tightening the writing—the paper has the potential to make a valuable contribution to the literature on social media, stigma, and health education.	I sincerely thank the reviewers for their valuable and constructive feedback. In response to the overall recommendation for major revisions, I have carefully reviewed and addressed each of the reviewers' comments in detail. Substantial revisions have been made throughout the manuscript, including the expansion of theoretical integration, deeper interpretation of findings, and improvements in clarity and coherence of the writing. All suggestions were thoughtfully considered, and the manuscript has been revised accordingly to enhance its academic rigor and potential contribution to the related literature.
