# Supplementary material for: Relationship between appearance-related social media consciousness and beliefs about obese persons among physical education teacher candidates
Source: PLoS One. 2026 Jul 8;21(7):e0350094. doi: 10.1371/journal.pone.0350094 (PMC13345289; doi:10.1371/journal.pone.0350094)
Supplement: Attachment — Submitted filename: Response to Reviewers Comments-Marc 25 2026.pdf [file pone.0350094.s006.pdf]

Dear Editor and Reviewers,

I sincerely thank you all for your constructive feedback, support, and invaluable comments, which were extremely helpful in revising the manuscript.

Below, I provide my responses to each of your comments.

Thanks to your guidance and suggestions, I believe the manuscript is now better aligned with the standards and expectations of PLOS ONE.

Manuscript ID: PONE-D-25-12083R1

Title: Relationship between appearance-related social media consciousness and beliefs about obese persons among physical education teacher candidates

Sincerely,

Canan Sayın Temur

Reviewer 1's Comments	Response to Reviewer 1's Comments
Beliefs about Obese Persons reliability (α = 0.57): Briefly discuss the implications of this low alpha for interpretation (attenuation of correlations, wider measurement error). If feasible, add an item-total table in the supplement and note whether any item(s) depressed alpha. If you choose not to alter the scale, state this is a known limitation and consider a sensitivity check (e.g., re-computing with one problematic item removed) reported in the supplement.	We thank the reviewer for this comment.<br>As suggested, we have added a brief discussion to the manuscript regarding the low reliability (α = 0.57) of the Beliefs about Obese Persons Scale.<br>We have noted that this may reduce the strength of correlations and increase measurement error.<br>We acknowledge this as a limitation and advise caution when interpreting the results. We have chosen to address this point in the text only, rather than providing an item-level table.<br>Page 12, Lines 1-6 and 20-23
Clarify BOP scoring text: Replace "adding 24 to the mean total score" with the exact scoring rule (e.g., reverse items 1, 3, 4, 5, 6, 8; sum all items; add 24 to shift the range to 0–48). This avoids confusion for replication.	Thank you for your valuable suggestion regarding the BAOP scoring.<br>We have clarified the scoring procedure in the manuscript: items 1, 3, 4, 5, 6, and 8 are reverse-coded, all 8 items are summed, and 24 is added to the total score to shift the range to 0–48, ensuring all scores are positive.<br>This revision should improve clarity and support accurate replication.<br>Page 12, Lines 11-14

Nonparametric summaries & effect sizes: Add IQRs to tables reporting medians. For Mann–Whitney r, present absolute values and provide a one-sentence interpretation (small/moderate) to emphasize practical significance, not just statistical significance.	We thank the reviewer for this valuable suggestion. Following your recommendation, we have added interquartile ranges (IQRs) to all tables reporting medians, and we have presented the absolute values of Mann–Whitney effect sizes (|r|), alongside a brief, one-sentence interpretation of their practical significance. These additions clarify the variability of the data and the magnitude of the observed effects, thereby enhancing the interpretability of our results.<br>Results section
**Reviewer 2's Comments**	**Response to Reviewer 2's Comments**
The manuscript continues to use normative language such as "slightly below average" or "just below the average" when describing beliefs about obese persons. These statements are not supported by external benchmarks or normative reference values. Please revise these to neutral, descriptive language, or clearly cite appropriate comparative data if such claims are to be retained.	Thank you for your careful evaluation of our manuscript and for your constructive and insightful comments.<br>In response to your comment about the use of normative expressions such as 'slightly below average' or 'just below the average' when describing beliefs about obese people, we have thoroughly revised the manuscript. Specifically, we have removed or replaced all such expressions with neutral, descriptive language that reflects the statistical findings directly, without implying external benchmarks.<br>As you correctly noted, the original phrasing was not supported by normative reference values, and we agree that it could potentially

	be misinterpreted.<br>The revised text now presents the results in a more objective, data-driven manner, focusing on reported scores and statistical comparisons rather than subjective qualifiers.<br>We believe that these changes have enhanced the scientific rigour and clarity of the manuscript.<br>Whole text
The internal consistency of the Beliefs about Obese Persons scale is low (Cronbach's α = 0.57) in the current sample. While this is transparently reported, its implications are not sufficiently discussed. Please add a brief but explicit discussion on how this low reliability may affect interpretation of the findings and caution readers accordingly.	Thank you for your valuable and constructive comment.<br>We agree that the relatively low internal consistency of the Beliefs about Obese Persons Scale in the current sample (Cronbach's α = 0.57) warrants careful consideration.<br>In the revised manuscript, we have explicitly addressed this issue, clarifying that the low reliability may increase measurement error and potentially reduce the strength of observed relationships, especially correlations.<br>Additionally, we emphasise that findings related to this scale should be interpreted with caution, and we have highlighted this limitation more clearly to ensure transparency for readers.<br>Pages 12, Lines 1-6 and 20-23

Although the limitations section appropriately notes the absence of multivariate analyses, some interpretations—particularly in the Discussion and Conclusion, remain somewhat stronger than warranted by bivariate analyses alone. Please further temper causal or explanatory language where appropriate.	We thank the reviewer for their valuable comment on how the findings should be interpreted in relation to the use of bivariate analyses.<br>In response, we have carefully revised the Discussion and Conclusion sections, tempering causal or explanatory language where appropriate.<br>Specifically, we have clarified that our cross-sectional design limits our ability to draw causal inferences, and emphasized that any observed differences or associations should be interpreted with caution.<br>Any statements that suggested causal relationships have been modified to indicate potential associations or tendencies rather than definitive conclusions.<br>We believe that these revisions address the reviewer's concerns while maintaining the integrity of the original findings.<br>Discussion and conclusion section
The response letter states that all underlying data will be made available in the Supporting Information, in line with PLOS ONE's data policy.<br>Please ensure that the revised submission clearly reflects this (and that the data files are indeed included), as this is a requirement for editorial compliance.	We have now included all the underlying data as Supporting Information files in the revised submission, ensuring compliance with PLOS ONE's data policy.

**Reviewer 3's Comments**	**Response to Reviewer 3's Comments**
Data collection tools, were they assessed for content validity and reliability assessment in particular to the population where they were administered. Comment on the applicability of these tools to the study participants.	Thank you for this valuable comment, which I really appreciate.<br><br>In response, we have revised the "Data Collection Tools" section to more clearly address the validity, reliability and applicability of the measurement instruments used in this study.<br><br>In particular, we have included statements highlighting that both scales have previously shown strong psychometric properties, including construct validity and internal consistency, in adolescent and young adult populations.<br><br>We also clarified that the study sample falls within a comparable age range. This supports the appropriateness of the instruments for the current population.<br><br>The study sample is physical education teacher candidates.<br><br>Moreover, we reported and interpreted the internal consistency coefficients obtained in this study explicitly.<br><br>The respective scale descriptions have been updated to reflect these revisions.<br><br>Page 10, Lines 22-23<br><br>Page 11, Lines 1-5<br><br>Page 12, Lines 1-6

Describe the study population, how they were selected, and where did they come from? Used convenient sampling, from where?	We appreciate the feedback from the reviewer. The Participants section of the manuscript has been updated to include details on the study location and sampling method. Researchers selected participants using a convenience sampling method, meaning that they included students based on their easy accessibility to them at Çukurova University, Adana, Turkey. Consequently, the sample may reflect characteristics specific to this university population, which could limit the generalisability of the findings.<br><br>Page 9, Lines 16-17
What was the level of significance considered in the analysis?	The level of significance considered in all statistical analyses was set at $p < 0.05$, and this is now explicitly stated in the 'Data Analysis' section of the manuscript.<br><br>Page 14, Lines 4-5
The author may include interquartile range for summary statistics to describe variability in the data.	We appreciate the reviewer's suggestion. We have calculated interquartile range (IQR) values for both scales and included them in Tables 1, 2, 3, and 4, which now provide a clearer picture of data dispersion alongside mean, standard deviation, and median values. These values were derived from the SPSS Descriptives and Percentiles outputs, and now provide a clearer picture of data dispersion alongside the mean, standard deviation and median.

Include reporting of median of each group in Kruskal wallis test results.	We would like to thank the reviewer for their helpful suggestion. In line with your recommendation, we have updated the Kruskal–Wallis test results table. In addition to the previously added interquartile range (IQR) values, we have included the median for each group to provide a more accurate description of the variability in the data.<br>Results section<br>Page 15-16
Suggests to not repeat in the text the numbers that are presented in the table, include spesification of direction of groups instead of repeating the numbers.	Thank you for your suggestion of avoiding the repetition of numeric values in the text. We have revised the results section in line with your advice, reporting only the direction and significance of the findings in the text, while keeping detailed statistics such as mean, standard deviation (SD), median, and interquartile range (IQR) in the tables.<br>Results section<br>Page 14-16
Suggest perform multiple comparison test to identify different groups.	Thank you for your suggestion. We conducted multiple comparison tests using Dunn's post hoc test with Bonferroni correction to determine which year groups were different. The results are presented in Tables 3 and 4.<br>Page 14-16
