# Supplementary material for: Relationship between appearance-related social media consciousness and beliefs about obese persons among physical education teacher candidates
Source: PLoS One. 2026 Jul 8;21(7):e0350094. doi: 10.1371/journal.pone.0350094 (PMC13345289; doi:10.1371/journal.pone.0350094)
Supplement: Attachment — Submitted filename: PLOS review.docx [file pone.0350094.s003.docx]

Relationship between appearance-related social media consciousness and beliefs about obese individuals among physical education teacher candidates

List of coments

1. Data collection tools, were they assessed for content validity and reliability assessment in particular to the population where they were administered.Comment on the applicability of these tools to the study participants.
2. Describe the study population, how they were selected, and where did they come from? Used convenient sampling, from where?
3. 3. What was the level of significance considered in the analysis?
4. 4. The author may include interquartile range for summary statistics to describe variability in the data
5. Include reporting of median og each group in Kruskal wallis test results
6. Suggests to not repeat in the text the numbers that are presented in the table, include spesification of direction of groups instead of repeating the numbers.
7. Suggest perform multiple comparison test to identify different groups.
