# Supplementary material for: Relationship between appearance-related social media consciousness and beliefs about obese persons among physical education teacher candidates
Source: PLoS One. 2026 Jul 8;21(7):e0350094. doi: 10.1371/journal.pone.0350094 (PMC13345289; doi:10.1371/journal.pone.0350094)
Supplement: Peer review history

# Peer review: Decision Letter 0

**DOI:** 10.1371/journal.pone.0350094.r001

**Authors:** Zypher Jude Regencia

10 Aug 2025

PONE-D-25-12083

Relationship between appearance-related social media consciousness and beliefs about obese individuals among physical education teacher candidates

PLOS ONE

Dear Dr. Sayın Temur,

Thank you for submitting your manuscript to PLOS ONE. After careful consideration, we feel that it has merit but does not fully meet PLOS ONE’s publication criteria as it currently stands. Therefore, we invite you to submit a revised version of the manuscript that addresses the points raised during the review process.

Please submit your revised manuscript by Sep 24 2025 11:59PM. If you will need more time than this to complete your revisions, please reply to this message or contact the journal office at plosone@plos.org. When you're ready to submit your revision, log on to https://www.editorialmanager.com/pone/ and select the 'Submissions Needing Revision' folder to locate your manuscript file.

Please include the following items when submitting your revised manuscript:

- A rebuttal letter that responds to each point raised by the academic editor and reviewer(s). You should upload this letter as a separate file labeled 'Response to Reviewers'.
- A marked-up copy of your manuscript that highlights changes made to the original version. You should upload this as a separate file labeled 'Revised Manuscript with Track Changes'.
- An unmarked version of your revised paper without tracked changes. You should upload this as a separate file labeled 'Manuscript'.

If you would like to make changes to your financial disclosure, please include your updated statement in your cover letter. Guidelines for resubmitting your figure files are available below the reviewer comments at the end of this letter.

If applicable, we recommend that you deposit your laboratory protocols in protocols.io to enhance the reproducibility of your results. Protocols.io assigns your protocol its own identifier (DOI) so that it can be cited independently in the future. For instructions see: https://journals.plos.org/plosone/s/submission-guidelines#loc-laboratory-protocols. Additionally, PLOS ONE offers an option for publishing peer-reviewed Lab Protocol articles, which describe protocols hosted on protocols.io. Read more information on sharing protocols at https://plos.org/protocols?utm_medium=editorial-email&utm_source=authorletters&utm_campaign=protocols.

We look forward to receiving your revised manuscript.

Kind regards,

Zypher Jude G. Regencia, Ph.D.

Academic Editor

PLOS ONE

Journal Requirements:

When submitting your revision, we need you to address these additional requirements.

1. Please ensure that your manuscript meets PLOS ONE's style requirements, including those for file naming. The PLOS ONE style templates can be found at

https://journals.plos.org/plosone/s/file?id=wjVg/PLOSOne_formatting_sample_main_body.pdf  and

https://journals.plos.org/plosone/s/file?id=ba62/PLOSOne_formatting_sample_title_authors_affiliations.pdf

2. In this instance it seems there may be acceptable restrictions in place that prevent the public sharing of your minimal data. However, in line with our goal of ensuring long-term data availability to all interested researchers, PLOS’ Data Policy states that authors cannot be the sole named individuals responsible for ensuring data access (http://journals.plos.org/plosone/s/data-availability#loc-acceptable-data-sharing-methods).

Data requests to a non-author institutional point of contact, such as a data access or ethics committee, helps guarantee long term stability and availability of data. Providing interested researchers with a durable point of contact ensures data will be accessible even if an author changes email addresses, institutions, or becomes unavailable to answer requests.

Before we proceed with your manuscript, please also provide non-author contact information (phone/email/hyperlink) for a data access committee, ethics committee, or other institutional body to which data requests may be sent. If no institutional body is available to respond to requests for your minimal data, please consider if there any institutional representatives who did not collaborate in the study, and are not listed as authors on the manuscript, who would be able to hold the data and respond to external requests for data access? If so, please provide their contact information (i.e., email address). Please also provide details on how you will ensure persistent or long-term data storage and availability.

3. Please include your full ethics statement in the ‘Methods’ section of your manuscript file. In your statement, please include the full name of the IRB or ethics committee who approved or waived your study, as well as whether or not you obtained informed written or verbal consent. If consent was waived for your study, please include this information in your statement as well.

4. If the reviewer comments include a recommendation to cite specific previously published works, please review and evaluate these publications to determine whether they are relevant and should be cited. There is no requirement to cite these works unless the editor has indicated otherwise.

[Note: HTML markup is below. Please do not edit.]

Reviewers' comments:

Reviewer's Responses to Questions

**Comments to the Author**

1. Is the manuscript technically sound, and do the data support the conclusions?

The manuscript must describe a technically sound piece of scientific research with data that supports the conclusions. Experiments must have been conducted rigorously, with appropriate controls, replication, and sample sizes. The conclusions must be drawn appropriately based on the data presented.

Reviewer #1: Yes

Reviewer #2: Partly

**********

2. Has the statistical analysis been performed appropriately and rigorously?

Reviewer #1: Yes

Reviewer #2: No

**********

3. Have the authors made all data underlying the findings in their manuscript fully available?

The PLOS Data policy requires authors to make all data underlying the findings described in their manuscript fully available without restriction, with rare exception (please refer to the Data Availability Statement in the manuscript PDF file). The data should be provided as part of the manuscript or its supporting information, or deposited to a public repository. For example, in addition to summary statistics, the data points behind means, medians and variance measures should be available. If there are restrictions on publicly sharing data—e.g. participant privacy or use of data from a third party—those must be specified.

Reviewer #1: No

Reviewer #2: No

**********

4. Is the manuscript presented in an intelligible fashion and written in standard English?

PLOS ONE does not copyedit accepted manuscripts, so the language in submitted articles must be clear, correct, and unambiguous. Any typographical or grammatical errors should be corrected at revision, so please note any specific errors here.

Reviewer #1: Yes

Reviewer #2: Yes

**********

5. Review Comments to the Author

Please use the space provided to explain your answers to the questions above. You may also include additional comments for the author, including concerns about dual publication, research ethics, or publication ethics. (Please upload your review as an attachment if it exceeds 20,000 characters)

Reviewer #1: This is a thoughtfully executed study on a topic of growing global relevance. It successfully addresses a research gap, contributes valuable cross-sectional data, and lays groundwork for further exploration into stigma and digital self-awareness in teacher education. While the analyses are basic and the conclusions somewhat cautious, the manuscript offers timely insights that merit dissemination. Observations and opportunities to improve the paper are as follows:

Introduction

The manuscript's objectives are clearly stated and relevant, particularly considering the WHO's projections on obesity prevalence and the pedagogical imperative for inclusive education. However, the introduction could benefit from a sharper statement of the core research question, ideally summarized at the end of the first section to enhance coherence.

Recommendation:

Clarify and explicitly state the research question/s at the end of the Introduction.

Condense the literature review to reduce redundancy and improve flow.

Methods

Observation:

Convenience sampling may introduce bias and limits generalizability. Cross-sectional design precludes causal inference. The study does not control for potential confounders such as BMI, personal history of dieting, or social media use frequency. Post-hoc testing lacks correction for multiple comparisons, increasing the risk of Type I error.

Recommendation:

The methods section should discuss these limitations more transparently, especially the implications of sampling bias and uncontrolled confounding variables.

Report effect sizes for all inferential tests and consider corrections for multiple comparisons.

Results/ Discussion

Observation:

The interpretations are consistent with the data and responsibly framed within the study's limitations. The authors rightly caution against overgeneralization and call for targeted training in PE curricula. Notably, they interpret the absence of gender differences and the significance of grade-level trends with nuance, acknowledging sociocultural factors and the potential role of educational exposure. However, the conclusion about "lower-than-average" beliefs and awareness is not empirically substantiated and seems speculative.

Recommendation:

Avoid drawing normative conclusions (e.g., that beliefs are "lower than average") unless benchmark data are cited. Emphasize the potential for curriculum interventions rather than assuming deficit thinking among participants.

Suggest future research directions involving longitudinal or qualitative methods to explore causality and context.

Reviewer #2: 1. Significance and Relevance

a. The topic is highly relevant, especially in the context of increasing body dissatisfaction and obesity stigma amplified by social media exposure. The focus on future PE teachers is well-justified as they play a key role in shaping student attitudes toward physical health and body image.

b. However, the manuscript misses an opportunity to deepen its theoretical and practical contributions. While the descriptive findings are useful, they are not sufficiently connected to broader frameworks such as social cognitive theory, stigma internalization, or health belief models.

2. Research Questions and Rationale

a. The study's objectives are clearly stated, but the rationale for exploring the relationship between appearance-related social media consciousness (ARSMC) and beliefs about obese persons (BOP) could be strengthened.

b. There is limited justification for why these two constructs should be correlated. While both relate to body image, a theoretical link (e.g., internalization of appearance ideals leading to weight stigma) would clarify expectations and better explain null results.

The manuscript might benefit from explicitly stating hypotheses or expected directions of relationships.

3. Sample and Generalizability

a. The study sample is limited to one university and selected via convenience sampling, which restricts external validity. This should be acknowledged more critically in the discussion section.

b. A sample of 153 participants is sufficient for nonparametric analysis but may be underpowered to detect small to moderate correlations. This limitation is not mentioned.

c. The demographic profile lacks important variables such as participants' BMI, frequency/type of social media use, and personal experience with weight stigma, which could all be relevant moderators or confounders.

4. Measures and Instrumentation

a. The ARSMC and BOP scales used are both validated and their Turkish adaptations are appropriate. The reported Cronbach’s alpha coefficients (0.94 and 0.85, respectively) indicate strong internal consistency.

b. It would be helpful to include more information about the factor structure or example items in the Results or Appendix for readers unfamiliar with these scales.

c. The BOP scale's scoring method (adding 24 to the sum of reverse-coded items) could be better explained, as this may not be intuitive to all readers.

5. Statistical Analysis

a. The use of nonparametric tests (Mann-Whitney U, Kruskal-Wallis, and Spearman’s rho) is justified due to the non-normal distribution of scores. However, the manuscript relies exclusively on bivariate analyses.

b. The absence of multivariate analyses is a missed opportunity. Simple adjustments for confounders such as age, gender, year level, or self-reported social media use could have added depth to the findings.

c. The decision not to use regression or structural equation modeling to explore mediation or moderation effects limits the interpretability of the null findings.

6. Results Interpretation

a. The findings are presented clearly, but the emphasis is on statistical significance rather than effect size or practical relevance. Reporting median differences, interquartile ranges, or even using boxplots could enhance the results section.

b. The null correlation between ARSMC and BOP is potentially meaningful and deserves deeper interpretation. Could it reflect a disconnect between internalized appearance concerns and external attitudes toward others?

c. The pattern of higher ARSMC and more positive beliefs among 4th-year students raises interesting possibilities. Have they received specific training that influenced their perceptions? Is there an educational progression effect?

7. Discussion and Integration with Literature

a. The discussion offers a useful summary of national and international studies, but it lacks a critical synthesis. Many citations are used descriptively without analyzing why findings converge or diverge.

b. The interpretation of null gender differences could benefit from engagement with recent debates on gendered body image expectations, especially in athletic or PE contexts.

c. The lack of a correlation between ARSMC and BOP should not be interpreted simply as "no relationship"; alternative explanations such as measurement limitations, range restriction, or unmeasured confounding should be acknowledged.

8. Limitations and Future Directions

a. Some limitations are mentioned, such as convenience sampling and small sample size, but these are treated briefly. A more reflective account of the study’s methodological constraints would strengthen the manuscript.

b. The recommendation to revise PE curricula is reasonable but would benefit from greater specificity. For example, what pedagogical approaches or training modules are proposed? Have such interventions been tested elsewhere?

c. Future research should consider a longitudinal design to examine how PE candidates' beliefs evolve over time and how professional training mediates these changes.

9. Writing Style and Structure

a. The manuscript is generally well-organized and readable. However, the introduction and discussion are quite long and at times repetitive. Several paragraphs could be condensed to improve focus and clarity.

b. Some terms (e.g., "beliefs about obese persons") are used frequently without variation; editorial revision could enhance linguistic variety and readability.

c. The manuscript would benefit from professional language editing to tighten the narrative, especially in the background and discussion sections.

10. Ethics and Data Availability

a. Ethical approval is clearly stated, and participant consent was obtained, which aligns with ethical standards.

b. The data availability statement ("available upon reasonable request") falls short of PLOS ONE’s expectations. Authors should consider uploading anonymized datasets to a public repository or providing more detailed access instructions.

Overall Recommendation: Major Revisions

This manuscript addresses an important and socially relevant issue, using validated instruments and appropriate analysis. However, the study design is limited in explanatory depth, and the theoretical integration is underdeveloped. With substantial revisions—particularly in expanding interpretation, integrating theory, and tightening the writing—the paper has the potential to make a valuable contribution to the literature on social media, stigma, and health education.

**********

6. PLOS authors have the option to publish the peer review history of their article (what does this mean?). If published, this will include your full peer review and any attached files.

If you choose “no”, your identity will remain anonymous but your review may still be made public.

**Do you want your identity to be public for this peer review?**  For information about this choice, including consent withdrawal, please see our Privacy Policy.

Reviewer #1: No

Reviewer #2: **Yes:** Dr. Zypher Jude G. Regencia

**********

[NOTE: If reviewer comments were submitted as an attachment file, they will be attached to this email and accessible via the submission site. Please log into your account, locate the manuscript record, and check for the action link "View Attachments". If this link does not appear, there are no attachment files.]

While revising your submission, please upload your figure files to the Preflight Analysis and Conversion Engine (PACE) digital diagnostic tool, https://pacev2.apexcovantage.com/. PACE helps ensure that figures meet PLOS requirements. To use PACE, you must first register as a user. Registration is free. Then, login and navigate to the UPLOAD tab, where you will find detailed instructions on how to use the tool. If you encounter any issues or have any questions when using PACE, please email PLOS at figures@plos.org. Please note that Supporting Information files do not need this step.

# Author comment: Author response to Decision Letter 1

**DOI:** 10.1371/journal.pone.0350094.r002

3 Sep 2025

1. Is the manuscript technically sound, and do the data support the conclusions?

The manuscript must describe a technically sound piece of scientific research with data that supports the conclusions. Experiments must have been conducted rigorously, with appropriate controls, replication, and sample sizes. The conclusions must be drawn appropriately based on the data presented.

Reviewer #1: Yes

Reviewer #2: Partly

I would like to express my sincere gratitude to the reviewers for their careful review and feedback.

This is a technically sound scientific study, and the data provided strongly support the reliability of the results. The study was meticulously planned with reproducibility in mind, and the sample size was determined in accordance with scientific standards. Transparency and objectivity were maintained when interpreting the results, using both quantitative data and visual tables. While Reviewer #1's positive assessment of 'Yes' confirms this, Reviewer #2's assessment of 'Partially' may indicate some shortcomings or areas requiring improvement.

A careful review of the entire research manuscript was therefore undertaken, concluding that the study is methodologically sound and that the data are robust enough to support the conclusions.

2. Has the statistical analysis been performed appropriately and rigorously?

Reviewer #1: Yes

Reviewer #2: No

Thank you for your valuable feedback regarding the statistical analysis. In response to your comment, I have revised the statistical section to enhance the rigor of our analysis. Specifically, I have calculated and reported the effect sizes for the Mann– Whitney U tests, as recommended for nonparametric comparisons. Additionally, for the Kruskal–Wallis tests that yielded statistically significant results, I have

conducted post-hoc pairwise comparisons using Dunn’s test with Bonferroni correction to control for multiple comparisons. These changes have been incorporated into method/data analysis and results section of the revised manuscript, and I believe they strengthen the statistical robustness of the findings.

3. Have the authors made all data underlying the findings in their manuscript fully available?

The PLOS Data policy requires authors to make all data underlying the findings described in their manuscript fully available without restriction, with rare exception (please refer to the Data Availability Statement in the manuscript PDF file). The data should be provided as part of the manuscript or its supporting information, or deposited to a public repository. For example, in addition to summary statistics, the data points behind means, medians and variance measures should be available. If there are restrictions on publicly sharing data—e.g. participant privacy or use of data from a third party—those must be specified.

Reviewer #1: No

Reviewer #2: No

Thank you for your suggestion. I confirm that all data underlying the findings of our manuscript will be made fully available in accordance with the PLOS ONE Data Availability Policy.

The complete dataset will be provided as part of the Supporting Information files.

4. Is the manuscript presented in an intelligible fashion and written in standard English?

PLOS ONE does not copyedit accepted manuscripts, so the language in submitted articles must be clear, correct, and unambiguous. Any typographical or grammatical errors should be corrected at revision, so please note any specific errors here.

Reviewer #1: Yes

Reviewer #2: Yes

I thank both reviewers for their careful assessment and positive feedback regarding the manuscript’s clarity and use of standard English (Reviewer #1: Yes; Reviewer #2:Yes).

I have thoroughly revised the manuscript from beginning to end, focusing on language, grammar, and typographical accuracy to ensure that the text is as clear, correct, and unambiguous as possible. All identified errors have been addressed in the revised version.

Reviewer #1: This is a thoughtfully executed study on a topic of growing global relevance. It successfully addresses a research gap, contributes valuable cross-sectional data, and lays groundwork for further exploration into stigma and digital self-awareness in teacher education. While the analyses are basic and the conclusions somewhat cautious, the manuscript offers timely insights that merit dissemination.

Observations and opportunities to improve the paper are as follows: Introduction

The manuscript's objectives are clearly stated and relevant, particularly considering the WHO's projections on obesity prevalence and the pedagogical imperative for inclusive education. However, the introduction could benefit from a sharper statement of the core research question, ideally summarized at the end of the first section to enhance coherence. Recommendation: Clarify and explicitly state the research

question/s at the end of the Introduction.

Condense the literature review to reduce redundancy and improve flow.

I would like to sincerely thank both reviewers. Their feedback was insightful and constructive. I have thoroughly revised the introduction section in response to the following points that were raised. I have rephrased the purpose statements at the end of the first section of the Introduction to make them clearer and more concise. This enhanced coherence and provide readers with a more focused understanding of the study's aims.

I have also made improvements to the literature review. I have removed references that are no longer needed and restructured the narrative to make it more reader-friendly. The context was made more succinct and relevant to the research objectives as a result.

Methods

Observation:

Convenience sampling may introduce bias and limits generalizability. Cross-sectional design precludes causal inference. The study does not

control for potential confounders such as BMI, personal history of dieting, or social media use frequency. Post-hoc testing lacks correction for

multiple comparisons, increasing the risk of Type I error.

Recommendation:

The methods section should discuss these limitations more transparently, especially the implications of sampling bias and uncontrolled confounding variables. Report effect sizes for all inferential tests and consider corrections for multiple comparisons.

I sincerely thank the reviewers for their valuable and constructive suggestions regarding the Methods section. I have carefully considered your feedback and made the necessary revisions to address the concerns.

I have expanded the Method/Participants and Limitations sections to more explicitly acknowledge the use of convenience sampling and the inherent limitations it poses in terms of potential selection bias and limited generalizability.

I have also clarified that the cross-sectional design precludes any causal inferences, and I have discussed how this affects the interpretation of the findings in both the Method/Research Design and Limitations sections.

Furthermore, I have acknowledged in the manuscript that important potential confounders—such as BMI, personal history of dieting, and frequency of social media use—were not controlled for. The implications of these uncontrolled variables have been discussed as a limitation.

Effect sizes have now been reported for all inferential statistical tests to provide a more comprehensive understanding of the magnitude of observed effects.

Additionally, I have revised the statistical analysis to include corrections for multiple comparisons in all post-hoc tests.

Specifically, the Bonferroni correction was applied to reduce the risk of Type I error. These revisions are reflected in the Methods, Results, and Limitations sections of the revised manuscript.

Results/ Discussion

Observation:

The interpretations are consistent with the data and responsibly framed within the study's limitations. The authors rightly caution against overgeneralization and call for targeted training in PE curricula. Notably, they interpret the absence of gender differences and the significance of grade-level trends with nuance, acknowledging sociocultural factors and the potential role of educational exposure. However, the conclusion about "lower-thanaverage" beliefs and awareness is not empirically substantiated and seems speculative.

Recommendation:

Avoid drawing normative conclusions (e.g., that beliefs are "lower than average") unless benchmark data are cited. Emphasize the potential for curriculum interventions rather than assuming deficit thinking among participants. Suggest future research directions involving longitudinal or qualitative methods to explore causality and context.

I sincerely thank the reviewers for their valuable and constructive suggestions. I have replaced normative expressions (such as stating beliefs are “lower than average”) with neutral, descriptive language. Where appropriate, I introduced benchmark data or referenced comparable studies to contextualize findings.

In the Discussion section, I have also added a dedicated paragraph outlining several future research directions, such as the use of longitudinal designs to better assess causal relationships, and the inclusion of qualitative methods to gain a deeper understanding of

participants' experiences and perspectives. In addition to suggestions for future studies, I have included practical implications of the

findings, offering recommendations that may inform interventions and practices in relevant applied settings.

Reviewer #2: 1. Significance and Relevance

a. The topic is highly relevant, especially in the context of increasing body dissatisfaction and obesity stigma amplified by social media exposure. The focus on future PE teachers is well-justified as they play a key role in shaping student attitudes toward physical health and body image. b. However, the manuscript misses an opportunity to deepen its theoretical and practical contributions. While the descriptive findings are useful, they are not sufficiently connected to broader frameworks such as social cognitive theory, stigma internalization, or health belief models.

I sincerely thank Reviewer #2 for highlighting the importance of deepening the manuscript’s theoretical and practical contributions. We have carefully addressed the feedback. I have enriched the Introduction by explicitly framing study findings within established theoretical models of Social Cognitive Theory (Bandura, 1986), Health Belief Model (Rosenstock, 1974), Stigma Internalization (Durso & Latner, 2008).

These additions aim to enhance the manuscript’s depth, ensuring it makes clearer contributions to both theory and practice.

2. Research Questions and Rationale

a. The study's objectives are clearly stated, but the rationale for exploring the relationship between appearance-related social media consciousness (ARSMC) and beliefs about obese persons (BOP) could be strengthened.

b. There is limited justification for why these two constructs should be correlated. While both relate to body image, a theoretical link (e.g., internalization of appearance ideals leading to weight stigma) would clarify expectations and better explain null results.

The manuscript might benefit from explicitly stating hypotheses or expected directions of relationships.

Thank you for the insightful comments. In response, the manuscript has been revised to strengthen the rationale for examining the relationship between appearance-related social media consciousness (ARSMC) and beliefs about obese persons (BOP). Theoretical links have been clarified, and objectives with expected directions of relationships have been explicitly stated to provide clearer guidance for the study.

3. Sample and Generalizability

a. The study sample is limited to one university and selected via convenience sampling, which restricts external validity. This should be acknowledged more critically in the discussion section.

b. A sample of 153 participants is sufficient for nonparametric analysis but may be underpowered to detect small to moderate correlations. This limitation is not mentioned.

c. The demographic profile lacks important variables such as participants' BMI, frequency/type of social media use, and personal experience with weight stigma, which could all be relevant moderators or confounders.

Thank you for these valuable comments. I acknowledge the limitations related to the use of a convenience sample from a single university. This limitation and its implications for the external validity and generalizability of the findings have been more critically discussed in the revised limitations section. I also recognize the absence of potentially relevant demographic variables such as participants’ BMI, the frequency and type of social media use, physical activity level, and personal experiences with weight stigma. These factors may serve as important moderators or confounders. I have addressed this omission in the limitations section and discussed how inclusion of these variables could inform future research.

4. Measures and Instrumentation

a. The ARSMC and BOP scales used are both validated and their Turkish adaptations are appropriate. The reported Cronbach’s alpha coefficients (0.94 and 0.85, respectively) indicate strong internal consistency.

b. It would be helpful to include more information about the factor structure or example items in the Results or Appendix for readers unfamiliar with these scales.

c. The BOP scale's scoring method (adding 24 to the sum of reverse-coded items) could be better explained, as this may not be intuitive to

all readers.

Thank you for your helpful comments. I appreciate your positive remarks regarding the validity and internal consistency of the ARSMC and BOP scales, as well as the appropriateness of their Turkish versions. To improve clarity for readers unfamiliar with these scales, I have expanded my descriptions of both instruments in the Methods section. I have provided additional information on the content and focus of each scale. Furthermore, example items from both the ARSMC and BOP scales have been added to give readers a clearer understanding of what the instruments measure.

5. Statistical Analysis

a. The use of nonparametric tests (Mann- Whitney U, Kruskal-Wallis, and Spearman’s rho) is justified due to the non-normal distribution of scores. However, the manuscript relies exclusively on bivariate analyses.

b. The absence of multivariate analyses is a missed opportunity. Simple adjustments for confounders such as age, gender, year level, or self-reported social media use could have added depth to the findings.

c. The decision not to use regression or structural equation modeling to explore mediation or moderation effects limits the interpretability of the null findings.

Thank you for your insightful comments regarding the statistical analyses. In response, I have calculated and reported effect sizes for the Mann–Whitney U tests to provide a clearer indication of the magnitude of observed effects. For the Kruskal–Wallis tests that yielded statistically significant results, I performed post-hoc pairwise comparisons using Dunn’s test with Bonferroni correction to account for multiple comparisons.

Regarding the concern about confounding variables, I acknowledge the limitation posed by not including multivariate analyses in the current study. I have added a discussion of potential confounders in the Method and Limitations section of the manuscript, highlighting how these uncontrolled variables may impact the findings and interpretation.

While regression or structural equation modeling approaches could offer deeper insights into mediation or moderation effects, these were beyond the scope of the current exploratory analysis.

6. Results Interpretation

a. The findings are presented clearly, but the emphasis is on statistical significance rather than effect size or practical relevance.

Reporting median differences, interquartile ranges, or even using boxplots could enhance the results section.

b. The null correlation between ARSMC and BOP is potentially meaningful and deserves deeper interpretation. Could it reflect a

disconnect between internalized appearance concerns and external attitudes toward others?

c. The pattern of higher ARSMC and more positive beliefs among 4th-year studen

Attachment Submitted filename: Response to Reviewers Comments.pdf

# Peer review: Decision Letter 1

**DOI:** 10.1371/journal.pone.0350094.r003

**Authors:** Zypher Jude Regencia

11 Mar 2026

PONE-D-25-12083R1

Relationship between appearance-related social media consciousness and beliefs about obese persons among physical education teacher candidates

PLOS One

Dear Dr. Sayın Temur,

Thank you for submitting your manuscript to PLOS ONE. After careful consideration, we feel that it has merit but does not fully meet PLOS ONE’s publication criteria as it currently stands. Therefore, we invite you to submit a revised version of the manuscript that addresses the points raised during the review process.

Please submit your revised manuscript by Apr 24 2026 11:59PM. If you will need more time than this to complete your revisions, please reply to this message or contact the journal office at plosone@plos.org. When you're ready to submit your revision, log on to https://www.editorialmanager.com/pone/ and select the 'Submissions Needing Revision' folder to locate your manuscript file.

Please include the following items when submitting your revised manuscript:

- A letter that responds to each point raised by the academic editor and reviewer(s). You should upload this letter as a separate file labeled 'Response to Reviewers'.

- A marked-up copy of your manuscript that highlights changes made to the original version. You should upload this as a separate file labeled 'Revised Manuscript with Track Changes'.

- An unmarked version of your revised paper without tracked changes. You should upload this as a separate file labeled 'Manuscript'.

-->If you would like to make changes to your financial disclosure, please include your updated statement in your cover letter. Guidelines for resubmitting your figure files are available below the reviewer comments at the end of this letter.

If applicable, we recommend that you deposit your laboratory protocols in protocols.io to enhance the reproducibility of your results. Protocols.io assigns your protocol its own identifier (DOI) so that it can be cited independently in the future. For instructions see: https://journals.plos.org/plosone/s/submission-guidelines#loc-laboratory-protocols. Additionally, PLOS ONE offers an option for publishing peer-reviewed Lab Protocol articles, which describe protocols hosted on protocols.io. Read more information on sharing protocols at https://plos.org/protocols?utm_medium=editorial-email&utm_source=authorletters&utm_campaign=protocols.

We look forward to receiving your revised manuscript.

Kind regards,

Zypher Jude G. Regencia, Ph.D.

Academic Editor

PLOS One

Journal Requirements:

If the reviewer comments include a recommendation to cite specific previously published works, please review and evaluate these publications to determine whether they are relevant and should be cited. There is no requirement to cite these works unless the editor has indicated otherwise.

Please review your reference list to ensure that it is complete and correct. If you have cited papers that have been retracted, please include the rationale for doing so in the manuscript text, or remove these references and replace them with relevant current references. Any changes to the reference list should be mentioned in the rebuttal letter that accompanies your revised manuscript. If you need to cite a retracted article, indicate the article’s retracted status in the References list and also include a citation and full reference for the retraction notice.

Additional Editor Comments:

Please note that I have acted as a reviewer for this manuscript, and you will find my comments below, under Reviewer 2.

[Note: HTML markup is below. Please do not edit.]

Reviewers' comments:

Reviewer's Responses to Questions

**Comments to the Author**

1. If the authors have adequately addressed your comments raised in a previous round of review and you feel that this manuscript is now acceptable for publication, you may indicate that here to bypass the “Comments to the Author” section, enter your conflict of interest statement in the “Confidential to Editor” section, and submit your "Accept" recommendation.

Reviewer #1: (No Response)

Reviewer #2: (No Response)

Reviewer #3: (No Response)

**********

2. Is the manuscript technically sound, and do the data support the conclusions?

The manuscript must describe a technically sound piece of scientific research with data that supports the conclusions. Experiments must have been conducted rigorously, with appropriate controls, replication, and sample sizes. The conclusions must be drawn appropriately based on the data presented.

Reviewer #1: Partly

Reviewer #2: Yes

Reviewer #3: Partly

**********

3. Has the statistical analysis been performed appropriately and rigorously?

Reviewer #1: Yes

Reviewer #2: Yes

Reviewer #3: (No Response)

**********

4. Have the authors made all data underlying the findings in their manuscript fully available?

The PLOS Data policy requires authors to make all data underlying the findings described in their manuscript fully available without restriction, with rare exception (please refer to the Data Availability Statement in the manuscript PDF file). The data should be provided as part of the manuscript or its supporting information, or deposited to a public repository. For example, in addition to summary statistics, the data points behind means, medians and variance measures should be available. If there are restrictions on publicly sharing data—e.g. participant privacy or use of data from a third party—those must be specified.

Reviewer #1: Yes

Reviewer #2: Yes

Reviewer #3: Yes

**********

5. Is the manuscript presented in an intelligible fashion and written in standard English?

PLOS ONE does not copyedit accepted manuscripts, so the language in submitted articles must be clear, correct, and unambiguous. Any typographical or grammatical errors should be corrected at revision, so please note any specific errors here.

Reviewer #1: Yes

Reviewer #2: Yes

Reviewer #3: Yes

**********

6. Review Comments to the Author

Please use the space provided to explain your answers to the questions above. You may also include additional comments for the author, including concerns about dual publication, research ethics, or publication ethics. (Please upload your review as an attachment if it exceeds 20,000 characters)

Reviewer #1: Thank you for the chance to re-review this paper. You responded substantively to our prior requests.

I only have a few minor recommendations:

BOP reliability (α = 0.57): Briefly discuss the implications of this low alpha for interpretation (attenuation of correlations, wider measurement error). If feasible, add an item-total table in the supplement and note whether any item(s) depressed alpha. If you choose not to alter the scale, state this is a known limitation and consider a sensitivity check (e.g., re-computing with one problematic item removed) reported in the supplement.

Clarify BOP scoring text: Replace “adding 24 to the mean total score” with the exact scoring rule (e.g., reverse items 1,3,4,5,6,8; sum all items; add 24 to shift the range to 0–48). This avoids confusion for replication.

Nonparametric summaries & effect sizes: Add IQRs to tables reporting medians. For Mann–Whitney r, present absolute values and provide a one-sentence interpretation (small/moderate) to emphasize practical significance, not just statistical significance.

Reviewer #2: Thank you for submitting the revised version of the manuscript. The revision shows substantial effort and meaningful engagement with the reviewers’ feedback, particularly in strengthening the statistical reporting, theoretical framing, and discussion. Most major concerns have been addressed satisfactorily. However, there are a few remaining points that need to be resolved before the manuscript can be considered for acceptance.

Points that still need attention

-The manuscript continues to use normative language such as “slightly below average” or “just below the average” when describing beliefs about obese persons. These statements are not supported by external benchmarks or normative reference values. Please revise these to neutral, descriptive language, or clearly cite appropriate comparative data if such claims are to be retained.

-The internal consistency of the Beliefs about Obese Persons scale is low (Cronbach’s α = 0.57) in the current sample. While this is transparently reported, its implications are not sufficiently discussed. Please add a brief but explicit discussion on how this low reliability may affect interpretation of the findings and caution readers accordingly.

-Although the limitations section appropriately notes the absence of multivariate analyses, some interpretations—particularly in the Discussion and Conclusion, remain somewhat stronger than warranted by bivariate analyses alone. Please further temper causal or explanatory language where appropriate.

-The response letter states that all underlying data will be made available in the Supporting Information, in line with PLOS ONE’s data policy. Please ensure that the revised submission clearly reflects this (and that the data files are indeed included), as this is a requirement for editorial compliance.

Reviewer #3: List of coments

1. Data collection tools, were they assessed for content validity and reliability

assessment in particular to the population where they were administered.Comment

on the applicability of these tools to the study participants.

2. Describe the study population, how they were selected, and where did they come

from? Used convenient sampling, from where?

3. 3. What was the level of significance considered in the analysis?

4. 4. The author may include interquartile range for summary statistics to describe

variability in the data

5. Include reporting of median of each group in Kruskal wallis test results

6. Suggests to not repeat in the text the numbers that are presented in the table,

include specification of direction of groups instead of repeating the numbers

**********

7. PLOS authors have the option to publish the peer review history of their article (what does this mean?). If published, this will include your full peer review and any attached files.

If you choose “no”, your identity will remain anonymous but your review may still be made public.

**Do you want your identity to be public for this peer review?**  For information about this choice, including consent withdrawal, please see our Privacy Policy.

Reviewer #1: **Yes:** Noriel P. Calaguas, PhD, MSHSA, RN, ACRN

Reviewer #2: No

Reviewer #3: **Yes:** Prof. Maria Lourdes Amarillo

**********

[NOTE: If reviewer comments were submitted as an attachment file, they will be attached to this email and accessible via the submission site. Please log into your account, locate the manuscript record, and check for the action link "View Attachments". If this link does not appear, there are no attachment files.]

To ensure your figures meet our technical requirements, please review our figure guidelines: https://journals.plos.org/plosone/s/figures

You may also use PLOS’s free figure tool, NAAS, to help you prepare publication quality figures: https://journals.plos.org/plosone/s/figures#loc-tools-for-figure-preparation.

NAAS will assess whether your figures meet our technical requirements by comparing each figure against our figure specifications.

Attachment Submitted filename: PLOS review.docx

Attachment Submitted filename: PLOS review feb20.docx

# Author comment: Author response to Decision Letter 2

**DOI:** 10.1371/journal.pone.0350094.r004

2 May 2026

Reviewer 1’s Comments

Beliefs about Obese Persons reliability (α = 0.57): Briefly discuss the implications of this low alpha for interpretation (attenuation of correlations, wider measurement error). If feasible, add an item-total table in the supplement and note whether any item(s) depressed alpha. If you choose not to alter the scale, state this is a known limitation and consider a sensitivity check (e.g., re-computing with one problematic item removed) reported in the supplement.

Response to Reviewer 1’s Comments

We thank the reviewer for this comment.

As suggested, we have added a brief discussion to the manuscript regarding the low reliability (α = 0.57) of the Beliefs about Obese Persons Scale.

We have noted that this may reduce the strength of correlations and increase measurement error.

We acknowledge this as a limitation and advise caution when interpreting the results. We have chosen to address this point in the text only, rather than providing an item-level table.

Page 12, Lines 1-6 and 20-23

Reviewer 1’s Comments

Clarify BOP scoring text: Replace “adding 24 to the mean total score” with the exact scoring rule (e.g., reverse items 1, 3, 4, 5, 6, 8; sum all items; add 24 to shift the range to 0–48). This avoids confusion for replication.

Response to Reviewer 1’s Comments

Thank you for your valuable suggestion regarding the BAOP scoring.

We have clarified the scoring procedure in the manuscript: items 1, 3, 4, 5, 6, and 8 are reverse-coded, all 8 items are summed, and 24 is added to the total score to shift the range to 0–48, ensuring all scores are positive.

This revision should improve clarity and support accurate replication.

Page 12, Lines 11-14

Reviewer 1’s Comments

Nonparametric summaries & effect sizes: Add IQRs to tables reporting medians. For Mann– Whitney r, present absolute values and provide a one-sentence interpretation (small/moderate) to emphasize practical significance, not just statistical significance.

Response to Reviewer 1’s Comments

We thank the reviewer for this valuable suggestion. Following your recommendation, we have added interquartile ranges (IQRs) to all tables reporting medians, and we have presented the absolute values of Mann–Whitney effect sizes (|r|), alongside a brief, one-sentence interpretation of their practical significance.

These additions clarify the variability of the data and the magnitude of the observed effects, thereby enhancing the interpretability of our results.

Results section

Reviewer 2’s Comments

The manuscript continues to use normative language such as “slightly below average” or “just

below the average” when describing beliefs about obese persons. These statements are not supported by external benchmarks or normative reference values. Please revise these to neutral, descriptive language, or clearly cite appropriate comparative data if such claims are to be retained.

Response to Reviewer 2’s Comments

Thank you for your careful evaluation of our manuscript and for your constructive and

insightful comments.

In response to your comment about the use of normative expressions such as 'slightly below average' or 'just below the average' when describing beliefs about obese people, we have thoroughly revised the manuscript. Specifically, we have removed or replaced all such expressions with neutral, descriptive language that reflects the statistical findings directly, without implying external benchmarks.

As you correctly noted, the original phrasing was not supported by normative reference values, and we agree that it could potentially be misinterpreted.

The revised text now presents the results in a more objective, data-driven manner, focusing on reported scores and statistical comparisons rather than subjective qualifiers.

We believe that these changes have enhanced the scientific rigour and clarity of the manuscript.

Whole text

Reviewer 2’s Comments

The internal consistency of the Beliefs about Obese Persons scale is low (Cronbach’s α = 0.57) in the current sample. While this is transparently reported, its implications are not sufficiently discussed. Please add a brief but explicit discussion on how this low reliability may affect interpretation of the findings and caution readers accordingly.

Response to Reviewer 2’s Comments

Thank you for your valuable and constructive comment. We agree that the relatively low internal consistency of the Beliefs about Obese Persons Scale in the current sample (Cronbach’s α = 0.57) warrants careful consideration. In the revised manuscript, we have explicitly addressed this issue, clarifying that the low reliability may increase measurement error and potentially reduce the strength of observed relationships, especially correlations.

Additionally, we emphasise that findings related to this scale should be interpreted with caution, and we have highlighted this limitation more clearly to ensure transparency for readers.

Pages 12, Lines 1-6 and 20-23

Reviewer 2’s Comments

Although the limitations section appropriately notes the absence of multivariate analyses, some interpretations—particularly in the Discussion and Conclusion, remain somewhat stronger than warranted by bivariate analyses alone. Please further temper causal or explanatory language where appropriate.

Response to Reviewer 2’s Comments

We thank the reviewer for their valuable comment on how the findings should be interpreted in relation to the use of bivariate analyses.

In response, we have carefully revised the Discussion and Conclusion sections, tempering causal or explanatory language where appropriate.

Specifically, we have clarified that our crosssectional design limits our ability to draw causal inferences, and emphasized that any observed differences or associations should be interpreted with caution.

Any statements that suggested causal relationships have been modified to indicate potential associations or tendencies rather than definitive conclusions.

We believe that these revisions address the reviewer’s concerns while maintaining the integrity of the original findings.

Discussion and conclusion section

Reviewer 2’s Comments

The response letter states that all underlying data will be made available in the Supporting Information, in line with PLOS ONE’s data policy.

Please ensure that the revised submission clearly reflects this (and that the data files are indeed included), as this is a requirement for editorial compliance.

Response to Reviewer 2’s Comments

We have now included all the underlying data as Supporting Information files in the revised submission, ensuring compliance with PLOS ONE’s data policy.

Reviewer 3’s Comments

Data collection tools, were they assessed for content validity and reliability assessment in particular to the population where they were administered. Comment on the applicability of these tools to the study participants.

Response to Reviewer 3’s Comments

Thank you for this valuable comment, which I really appreciate.

In response, we have revised the "Data Collection Tools" section to more clearly address the validity, reliability and applicability of the measurement instruments used in this study.

In particular, we have included statements highlighting that both scales have previously shown strong psychometric properties, including construct validity and internal consistency, in adolescent and young adult populations.

We also clarified that the study sample falls within a comparable age range. This supports the appropriateness of the instruments for the current population.

The study sample is physical education teacher candidates.

Moreover, we reported and interpreted the internal consistency coefficients obtained in this study explicitly.

The respective scale descriptions have been updated to reflect these revisions.

Page 10, Lines 22-23

Page 11, Lines 1-5

Page 12, Lines 1-6

Reviewer 3’s Comments

Describe the study population, how they were selected, and where did they come from? Used convenient sampling, from where?

Response to Reviewer 3’s Comments

We appreciate the feedback from the reviewer. The Participants section of the manuscript has been updated to include details on the study location and sampling method. Researchers selected participants using a convenience sampling method, meaning that they included students based on their easy accessibility to them at Çukurova University, Adana, Turkey. Consequently, the sample may reflect characteristics specific to this university population, which could limit the generalisability of the findings.

Page 9, Lines 16-17

Reviewer 3’s Comments

What was the level of significance considered in the analysis?

Response to Reviewer 3’s Comments

The level of significance considered in all statistical analyses was set at p < 0.05, and this is now explicitly stated in the 'Data Analysis' section of the manuscript.

Page 14, Lines 4-5

Reviewer 3’s Comments

The author may include interquartile range for summary statistics to describe variability in the data.

Response to Reviewer 3’s Comments

We appreciate the reviewer’s suggestion. We have calculated interquartile range (IQR) values for both scales and included them in Tables 1, 2, 3, and 4, which now provide a clearer picture of data dispersion alongside mean, standard deviation, and median values. These values were derived from the SPSS Descriptives and Percentiles outputs, and now provide a clearer picture of data dispersion alongside the mean, standard deviation and median.

Reviewer 3’s Comments

Include reporting of median of each group in Kruskal wallis test results.

Response to Reviewer 3’s Comments

We would like to thank the reviewer for their helpful suggestion. In line with your recommendation, we have updated the Kruskal–Wallis test results table. In addition to the previously added interquartile range (IQR) values, we have included the median for each group to provide a more accurate description of the variability in the data.

Results section

Page 15-16

Reviewer 3’s Comments

Suggests to not repeat in the text the numbers that are presented in the table, include spesification of direction of groups instead of repeating the numbers.

Response to Reviewer 3’s Comments

Thank you for your suggestion of avoiding the repetition of numeric values in the text.

We have revised the results section in line with your advice, reporting only the direction and significance of the findings in the text, while keeping detailed statistics such as mean, standard deviation (SD), median, and interquartile range (IQR) in the tables.

Results section

Page 14-16

Reviewer 3’s Comments

Suggest perform multiple comparison test to identify different groups.

Response to Reviewer 3’s Comments

Thank you for your suggestion. We conducted multiple comparison tests using Dunn's post hoc test with Bonferroni correction to determine which year groups were different. The results are presented in

Tables 3 and 4.

Page 14-16

Attachment Submitted filename: Response to Reviewers Comments-Marc 25 2026.pdf

# Editor report: Decision Letter 2

**DOI:** 10.1371/journal.pone.0350094.r005

**Authors:** Zypher Jude Regencia

10 May 2026

<p>Relationship between appearance-related social media consciousness and beliefs about obese persons among physical education teacher candidates

PONE-D-25-12083R2

Dear Dr. Temur,

We’re pleased to inform you that your manuscript has been judged scientifically suitable for publication and will be formally accepted for publication once it meets all outstanding technical requirements.

Within one week, you’ll receive an e-mail detailing the required amendments. When these have been addressed, you’ll receive a formal acceptance letter and your manuscript will be scheduled for publication.

An invoice will be generated when your article is formally accepted. Please note, if your institution has a publishing partnership with PLOS and your article meets the relevant criteria, all or part of your publication costs will be covered. Please make sure your user information is up-to-date by logging into Editorial Manager at Editorial Manager® and clicking the ‘Update My Information' link at the top of the page. For questions related to billing, please contact billing support.

If your institution or institutions have a press office, please notify them about your upcoming paper to help maximize its impact. If they’ll be preparing press materials, please inform our press team as soon as possible -- no later than 48 hours after receiving the formal acceptance. Your manuscript will remain under strict press embargo until 2 pm Eastern Time on the date of publication. For more information, please contact onepress@plos.org.

Kind regards,

Zypher Jude G. Regencia, Ph.D.

Academic Editor

PLOS One

Additional Editor Comments (optional):

Reviewers' comments:

# Editor report: Acceptance letter

**DOI:** 10.1371/journal.pone.0350094.r006

**Authors:** Zypher Jude Regencia

PONE-D-25-12083R2

PLOS One

Dear Dr. Sayın Temur,

I'm pleased to inform you that your manuscript has been deemed suitable for publication in PLOS One. Congratulations! Your manuscript is now being handed over to our production team.

At this stage, our production department will prepare your paper for publication. This includes ensuring the following:

* All references, tables, and figures are properly cited

* All relevant supporting information is included in the manuscript submission,

* There are no issues that prevent the paper from being properly typeset

You will receive further instructions from the production team, including instructions on how to review your proof when it is ready. Please keep in mind that we are working through a large volume of accepted articles, so please give us a few days to review your paper and let you know the next and final steps.

Lastly, if your institution or institutions have a press office, please let them know about your upcoming paper now to help maximize its impact. If they'll be preparing press materials, please inform our press team within the next 48 hours. Your manuscript will remain under strict press embargo until 2 pm Eastern Time on the date of publication. For more information, please contact onepress@plos.org.

You will receive an invoice from PLOS for your publication fee after your manuscript has reached the completed accept phase. If you receive an email requesting payment before acceptance or for any other service, this may be a phishing scheme. Learn how to identify phishing emails and protect your accounts at https://explore.plos.org/phishing.

If we can help with anything else, please email us at customercare@plos.org.

Thank you for submitting your work to PLOS ONE and supporting open access.

Kind regards,

PLOS ONE Editorial Office Staff

on behalf of

Dr. Zypher Jude G. Regencia

Academic Editor

PLOS One
